# Hyperbolic disc embedding of functional human brain connectomes using resting-state fMRI

**DOI:** 10.1162/netn_a_00243

**Published:** 2022-07-01

**Authors:** Wonseok Whi, Seunggyun Ha, Hyejin Kang, Dong Soo Lee

**Affiliations:** Department of Molecular Medicine and Biopharmaceutical Sciences, Seoul National University, Seoul, South Korea; Department of Nuclear Medicine, Seoul National University and Seoul National University Hospital, Seoul, South Korea; Division of Nuclear Medicine, Department of Radiology, Seoul St. Mary's Hospital, Catholic University of Korea, Seoul, South Korea; Biomedical Research Institute, Seoul National University Hospital, Seoul, South Korea; Medical Research Center, Seoul National University, Seoul, South Korea

**Keywords:** Functional brain network, Hyperbolic geometry, Hyperbolic embedding, 𝕊^1^/ℍ^2^ model

## Abstract

The brain presents a real complex network of modular, small-world, and hierarchical nature, which are features of non-Euclidean geometry. Using resting-state functional magnetic resonance imaging, we constructed a scale-free binary graph for each subject, using internodal time series correlation of regions of interest as a proximity measure. The resulting network could be embedded onto manifolds of various curvatures and dimensions. While maintaining the fidelity of embedding (low distortion, high mean average precision), functional brain networks were found to be best represented in the hyperbolic disc. Using the 𝕊^1^/ℍ^2^ model, we reduced the dimension of the network into two-dimensional hyperbolic space and were able to efficiently visualize the internodal connections of the brain, preserving proximity as distances and angles on the hyperbolic discs. Each individual disc revealed relevance with its anatomic counterpart and absence of center-spaced node. Using the hyperbolic distance on the 𝕊^1^/ℍ^2^ model, we could detect the anomaly of network in autism spectrum disorder subjects. This procedure of embedding grants us a reliable new framework for studying functional brain networks and the possibility of detecting anomalies of the network in the hyperbolic disc on an individual scale.

## Author Summary

Human brain networks possess modular, small-world, and hierarchical natures, which necessitate the inspection of network in a basis of non-Euclidean geometry. In this work, using the time series correlation of BOLD signals from resting-state functional magnetic resonance imaging, we constructed a scale-free network for each individual subject and embedded the networks into hyperbolic discs of the 𝕊^1^/ℍ^2^ model. The embedding procedure showed reproducibility, and the embedded discs showed relevance with their anatomical counterparts. Then, we assessed the anomaly of brain networks in subjects with autism spectrum disorder by comparing hyperbolic distances of edges. This procedure leads us to a new method of reproducible visualization of functional brain networks and the possibility of detecting anomalies of internodal pathways in the brain network of a diseased subject, on an individual scale.

## INTRODUCTION

Significant progress has been achieved over the last decades in unveiling the structure and mechanism of the human brain, which is one of the most complicated systems of nature. In particular, studies on the network geometry, which offer a new perspective on the framework of structural brain networks (Betzel et al., 2014; Boguñá et al., 2021), or connectomes, have revealed that structural brain networks share certain topological properties such as modularity (Meunier et al., 2010), small-world (Bassett & Bullmore, 2017), and heavy-tailed degree distribution (Gastner & Ódor, 2016).

However, Euclidean geometry, which serves as a standard framework of investigation in our physical reality, does not seem to suffice to explain the observed connectivity between brain regions, functional as well as structural. Instead, an increasing amount of evidence suggests that the geometry of negative curvature, that is, hyperbolic geometry, serves as a more appropriate explanatory platform of the above-mentioned topologic features of the structural brain networks that dominates the formation of the network and the probability of connections between the brain regions (Allard & Serrano, 2020; Tadić et al., 2019).

Since the functional brain network is closely related to the connectomes (Markram et al., 2015; Sporns, 2013; Stam et al., 2016), we assume that the functional brain network best fits with the hyperbolic geometry, which is regarded as the geometric representation of tree or tree-like graphs with hierarchies (Clauset et al., 2008). However, tree-like graphs could also have a cycle or torus-like structures therein and necessitate the trial-fitting to space of positive or zero curvatures. As hyperbolic, spherical, and Euclidean space can be combined by product to represent real complex graphs, they can now be tested for their feasibility of fitting to the model of a product of spaces with various negative and/or positive curvatures. The only reserve is to find the model space of appropriate dimension and curvature to fit the real complex networks.

By this means, we later show that we successfully embedded the functional brain graphs observed on resting-state functional magnetic resonance imaging (rs-fMRI) to be fit to the two-dimensional hyperbolic plane (disc) with better fidelity compared with other product manifolds of low dimension. In this study, we used the 𝕊^1^/ℍ^2^ model, the latter of which is purely geometric, well known to reveal the hidden geometry of many other real complex networks of non-Euclidean nature, such as internet connection (Boguñá et al., 2010), world trade web (García-Pérez et al., 2016), or even the structural brain network (Allard & Serrano, 2020; Tadić et al., 2019).

When we adopted the binary graph obtained by rs-fMRI as an input to the 𝕊^1^/ℍ^2^ model, we could fit the nodes to their optimal positions on the hyperbolic disc proposed by Papadopoulos et al. (2012) and further publicized as Mercator (García-Pérez et al., 2019). The position of the nodes could be used to calculate the hyperbolic distance between each node, which serves as a unique variable for determining the connection probability between the nodes on this disc (Serrano et al., 2008). We used the distance matrix for comparison of the proximity between brain region pairs across individuals with autism spectrum disorder (ASD) and a control group to see whether the method is applicable to real cases of diseased subjects.

## METHODS

### Subjects

#### Human Connectome Project (HCP) dataset.

To investigate the general characteristics of the functional brain network, we used the rs-fMRI datasets from healthy adults of the Human Connectome Project (HCP) S1200 release. Participants were between 22 and 35 years of age at the time of recruitment and did not have any documented history of psychiatric, neurological, or medical disorders known to influence brain function. A more detailed description of the inclusion and exclusion criteria for HCP is shown in the literature (Van Essen et al., 2012). The sample of participants included 180 subjects (mean age = 29.1, *SD* = 3.4), with 76 male and 104 female subjects.

#### Autism Brain Imaging Data Exchange (ABIDE) dataset.

For the investigation of the characteristics of subjects diseased with ASD, we made use of 247 resting-state fMRI datasets from Autism Brain Imaging Data Exchange (ABIDE) I datasets, 113 from diseased subjects, and 134 from the control group. We selected data from the same age group as that of HCP datasets, whose age ranged between 20 and 35 years of age in the control group (mean age = 26.8, *SD* = 3.8), and 20 to 39 years of age in the diseased group (mean age = 27.9, *SD* = 4.9). A more detailed description of the subject information and image acquisition protocol is described in the literature (Di Martino et al., 2017).

### Functional Connectivity Analysis

We parcellated the whole brain in two scopes: (a) 274 regions of interest (ROIs) using the human Brainnetome Atlas (Fan et al., 2016). All of the 274 sub-ROIs were included in the analysis. Detailed information on ROIs, including full name and abbreviations, are available at https://atlas.brainnetome.org/. (b) Cubic isotropic voxels of 6 × 6 × 6 mm^3^ size, with a total number of 5,937 voxels. The voxel-scope analysis was restricted to 10 randomly selected cases of the HCP dataset because of long computation time.

Spontaneous fluctuations were characterized by the variance of the population σ^2^(*X*) of the blood oxygen level–dependent (BOLD) signal of fMRI time series X (Materna et al., 2008). For the BOLD-fMRI time series X = (X_1_, …, X_*N*_) of a given ROI, the variance was computed by the sample variance ${\hat{\sigma }}^{2}\left(X\right)$,

$${\hat{\sigma }}^{2}\left(X\right)=\frac{1}{N-1}\sum_{i=1}^{N}{\left(X_{i}-\bar{X}\right)}^{2},$$ (1)

where $\bar{X}$ denotes the sample mean of *X*. Functional connectivity was assessed by the Pearson correlation coefficient ρ:

$$\rho \left(X,Y\right)=\frac{Cov\left(X,Y\right)}{\sigma \left(X\right)\sigma \left(Y\right)},$$ (2)

where *Y* stands for another BOLD-fMRI time series. For a pair of BOLD-fMRI time series X = (X_1_, …, X_*N*_) and Y = (Y_1_, …, Y_*N*_), $\hat{\rho }$ was estimated by the sample Pearson correlation coefficient $\hat{\rho }$:

$$\hat{\rho }\left(X,Y\right)=\frac{1}{N-1}\sum_{i=1}^{N}\frac{\left(X_{i}-\bar{X}\right)\left(Y_{i}-\bar{Y}\right)}{\hat{\sigma }\left(X\right)\hat{\sigma }\left(Y\right)}.$$ (3)

From the Pearson correlation coefficients, we obtained a square matrix of Pearson correlation coefficients for each of the subjects. The connectivity matrix that consists of absolute values of both positive and negative correlation coefficients was used to establish a binary graph from the network. We tried multiple threshold values for each group analysis and compared the degree distribution and the number of nodes belonging to the largest connected component. We determined single fixed threshold value for each group (0.36 for the HCP dataset and 0.40 for ABIDE dataset) by considering two factors, by reviewing networks with threshold of 0.15 to 0.65, with intervals of 0.01: (a) Degree distribution of the network, determined by researchers if it follows power law degree distribution. (b) We wanted the network to include at least 95% (261 nodes, in case of 274 node networks in this work) of nodes of the original network. By these two factors, we have set the window for each subject (Supplementary Figure 1 in the Supporting Information) and set the single threshold value (for the sake of comparability) for each group that meets the most windows. Consequently, we constructed an unweighted, undirected graph for each subject by applying the threshold to the coefficient matrix.

### Embedding the Network Into Manifolds of Various Curvatures and Dimensions

An embedding of the network is assigning nodes to representative low-dimensional space, which effectively preserves the network structure (Cui et al., 2018). The analytical meaning of embedding is the mapping *f* : *U* → *V* for spaces *U*, *V* with distances *d*_*U*_, *d*_*V*_. We can measure the quality of embeddings with fidelity measures. The standard metric for graph embeddings is distortion *D* (Sala et al., 2018). For an *n*-point embedding,

Df=1n2∑u,v∈U:u≠vdVfufv−dUuvdUuv. (4)

The best distortion is *D*(*f*) = 0, preserving the edge lengths exactly. Also, note that *D*(*f*) can be larger than 1. This is a *global* metric, as it directly depends on the value of distances rather than on the local relationships, or ranks, between distances.

Preceding work by Nickel and Kiela (2017) suggests mean average precision (mAP) for another measure. For a graph *G* = (*V*, *E*), let *a* ∈ *V* have neighborhood 𝒩_*a*_ = {*b*_1_, *b*_2_, ⋯, *b*_*deg*(*a*)_}, where *deg*(*a*) denotes the degree of *a*. In the embedding *f*, consider the points closest to *f*(*a*), and define *R*_*a*,*b*_*i*__ to be the smallest set of such points that contains *b*_*i*_, that is, *R*_*a*,*b*_*i*__ is the smallest set of nearest points required to retrieve the *i*th neighbor of *a* in *f* (García-Pérez et al., 2020). Then, the mAP is defined to be

$$\begin{array}{l} mAP\left(f\right)=\frac{1}{\left|V\right|}\sum_{a\in V}\frac{1}{\left|{𝒩}_{a}\right|}\sum_{i=1}^{\left|{𝒩}_{a}\right|}\text{Precision}\left(R_{a,b_{i}}\right)\\ \;=\frac{1}{\left|V\right|}\sum_{a\in V}\frac{1}{\text{deg}\left(a\right)}\sum_{i=1}^{\left|{𝒩}_{a}\right|}\frac{\left|{𝒩}_{a}\cap R_{a,b_{i}}\right|}{\left|R_{a,b_{i}}\right|}. \end{array}$$ (5)

Note that mAP(*f*) *≤* 1. The equal sign is the best case that preserves the rank of distances between the nodes. The mAP is not concerned with the exact value of underlying distances but only with the relative ranks between the distances of immediate neighbors. This is a *local* metric.

To compute the quality of embeddings, we implemented the method proposed by Gu et al. (Chami et al., 2019; Gu et al., 2018; available at https://github.com/HazyResearch/hyperbolics), which optimizes the placement of points using an auxiliary loss function1, which utilizes the average distortion. To optimize the loss function, an algorithm using Riemannian stochastic gradient descent (RSGD), a standard Riemannian optimization method, was used. The algorithm first computed the Euclidean gradient of ambient space of embedding and converted it to the Riemannian gradient by Riemannian correction.

### Embedding Onto Hyperbolic Discs of the 𝕊^1^/ℍ^2^ Model

The binary graph from each patient was then embedded into latent hyperbolic geometry of the 𝕊^1^/ℍ^2^ geometric network model. In the model, the connection probability between two nodes *i* and *j* is determined by the hyperbolic distance between two nodes:

$$p_{ij}=\frac{1}{1+e^{\frac{\beta }{2}\left(d_{ij}-\hat{R}\right)}},$$ (6)

where *d*_*ij*_ is the hyperbolic distance, which has a good approximation:

$$d_{ij}≅r_{i}+r_{j}+2\;\ln\frac{\Delta \theta_{ij}}{2}+O\left(\frac{1}{r}\right),$$ (7)

where β is the clustering coefficient of the network and $\hat{R}$ is the outermost radial coordinate among the embedded nodes. We described the details of the 𝕊^1^/ℍ^2^ geometric model in Supplementary Note 1 in the Supporting Information. To find the most appropriate geometric object (i.e., a hyperbolic disc) that is most likely to generate the binary graph we made, we implemented Mercator, software introduced by García-Pérez et al. (2019; available at https://github.com/networkgeometry/mercator), which assumed that the structure of networks could be described by the 𝕊^1^/ℍ^2^ geometric model. We embedded an undirected, unweighted adjacency matrix for each subject onto a hyperbolic disc. In other words, we searched a set of angular coordinates for *N* nodes (r_1_, θ_1_), (r_2_, θ_2_), ⋯, (r_*N*_, θ_*N*_) and the clustering coefficient β on a hyperbolic disc.

We have shown the robustness of results over different thresholds in Supplementary Note 2. We also conducted an experiment with only positively correlated pairs of nodes, with the results shown in Supplementary Note 3 (see the Supporting Information).

### Comparison With ICA-Driven Results

To investigate the compatibility of the embedding with other established methods, resting-state networks determined from group independent component analysis (ICA) (van de Ven et al., 2004) were plotted on the individual hyperbolic discs for the voxel data. Each independent component was thresholded by a z-score larger than 6. As an ROI-derived correlation matrix was embedded onto 𝕊^1^/ℍ^2^ modeled hyperbolic discs, a voxel-derived correlation matrix on rs-fMRI was embedded onto hyperbolic discs and colored for the lobes used in ROI lobe coloring (Supplementary Figure 2) and also each independent component such as the default mode network (DMN), salience network (SN), visual network (VN), and others in voxel independent component coloring (Supplementary Figure 3).

### Reproducibility of Embedding in an Individual

We computed the hyperbolic distance between ROIs over multiple performances of embedding for one representative case and computed the coefficient of variance (CV) for distance analog, which is correlated with connection probability,

$$D_{ij}=e^{\frac{\left(d_{ij}-\hat{R}\right)}{2}},$$ (8)

which appears in the denominator of the right term of Equation 6 when we assume β equals 1 and is a simple increasing function *d*_*ij*_, for each pair of nodes. We computed the connection probability of edges and the CV value of distance analog over the repeated embedding of 100 times (Supplementary Figure 4). Then, to assess the arbitrariness of position of certain nodes over repeated instances of embedding, for each node of the network we again computed the average value of CV calculated for all edges connected to the node. Each node was colored according to the node’s average CV value on the hyperbolic disc of any one embedded disc among 100 embedded discs of repetition. An example was shown in Figure 6B.

### Detection of Abnormality of Hyperbolic Distance in the Diseased Subjects Compared With Controls

To find the abnormal pathway of the functional brain network in ASD group subjects, we used the ABIDE dataset and compared the distance matrices resulting from the 𝕊^1^/ℍ^2^ model embedding, according to the following process.

After embedding the individual networks according to the 𝕊^1^/ℍ^2^ hyperbolic embedding in the ROI scale, we computed the hyperbolic distance *d*_*ij*_ determined by Equation 7 between each pair of ROIs for each individual subject in the disease group and the control subjects, that is, individual to group comparison. We set the distribution of hyperbolic distances for each edge in the control group dataset. For each ASD group subject, we considered significant edges with higher/lower hyperbolic distances seen on the 𝕊^1^/ℍ^2^ models.

To compare the results with those from the traditional correlation study, we conducted a similar comparison by lower/higher correlation values between an individual and the control group. We selected edges of which hyperbolic distance was located in the upper or lower 2.5% of the distribution. In the control group, hyperbolic distances and correlation values for each edge were tested for normality (*P* < 0.05). Multiple comparison correction was not performed.

## RESULTS

### Distribution of Time Series Correlation in rs-fMRI

**Figure 1.**
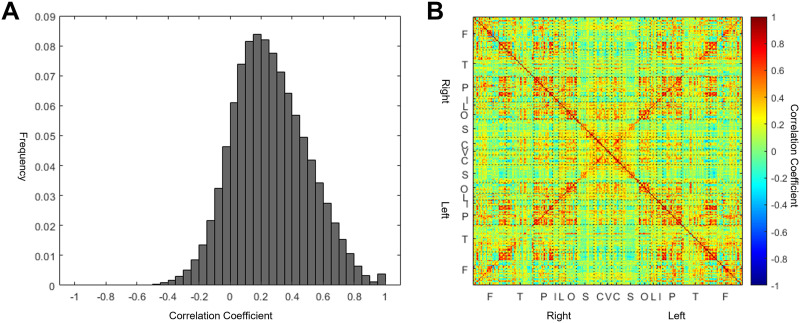
Distributions of time series correlation of resting-state fMRI signal. (A) Histogram of Pearson's correlation from all edges of 180 Human Connectome Project (HCP) subjects. The horizontal axis is the value of the Pearson’s correlation, while the vertical axis denotes the frequency of the value. The correlation is computed by $\hat{\rho }\left(X,Y\right)=\frac{1}{N-1}\sum_{i=1}^{N}\frac{\left(X_{i}-\bar{X}\right)\left(Y_{i}-\bar{Y}\right)}{\hat{\sigma }\left(X\right)\hat{\sigma }\left(Y\right)}$. A positive correlation between nodes was dominant in healthy adults, while a small portion of edges showed a negative correlation. Overall, 6.70% of edges had a higher absolute value than the threshold and were considered to be connected. (B) A matrix view of one representative subject. Symbols on each axis denote the anatomic lobe of the nodes. F = frontal, T = temporal, P = parietal, I = insula, L = limbic, O = occipital, S = subcortical, C = cerebellar, V = vermis.

The distribution of the value of correlation coefficients of $\frac{274\times 273}{2}$ node pairs is shown in (Figure 1A). Overall, a positive correlation was dominant among edges from the healthy young adults, while a small portion of edges showed a negative correlation. A detailed visualization of the correlation matrix from a representative case is shown in (Figure 1B). We regarded considered that a certain degree of anticorrelation between brain regions also suggested connection and took the absolute value of the correlation coefficient for thresholding the network.

### Determining the Threshold of Correlation for Binary Network Composition

To form the binarized adjacency matrix for each fMRI image, we applied various threshold values to the correlation matrix in order to choose the binary network that satisfies the scale-free property. The chosen threshold yielded a degree distribution that fit well to a linear plot on log-log relations between degree and degree frequency and included most of the nodes within the largest connected component of the resulting binary network. After we investigated the degree distribution of the graph and the average size of the largest connected component, we chose the threshold for individuals and finally the entire population. When we applied a higher threshold, more nodes without any connection were excluded from the component. On the other hand, when we lowered the threshold, some of the networks violated the scale-freeness, which we have excluded for conditioning for the later embedding procedure.

**Figure 2.**
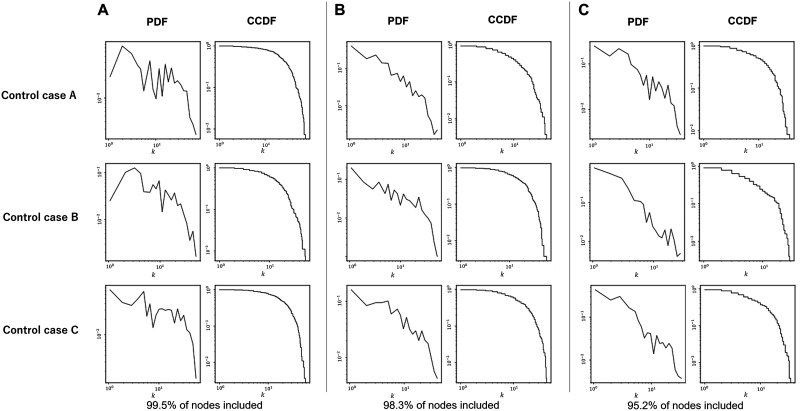
Determining the threshold of correlation for network composition. Probability distribution function (PDF) and complementary cumulative distribution function (CCDF) of degree distribution from three representative subjects from the ABIDE dataset. The threshold is set as (A) 0.35, (B) 0.40, and (C) 0.45. (A) With a threshold value of 0.35, in which most of the nodes are preserved, the degree distribution forms a peaked curve, which violates the scale-freeness of the network. (B) With a threshold value of 0.40, most of the nodes are preserved, while the degree distribution forms a relatively straight-downward line. (C) With a higher threshold value of 0.45, 4.8% of nodes, an average of 13 nodes per network are excluded from the largest component, which we considered inappropriate for a whole-brain analysis. Therefore, the threshold value was determined to be 0.40.

Since we wanted to retain as many nodes as possible in every case, we applied the highest threshold possible while the scale-freeness of the network is valid. These procedures are shown in Figure 2 for three cases of control subjects from the ABIDE dataset. With a threshold value of 0.35 (Figure 2A), in which most of the nodes are preserved, the degree distribution forms a peaked curve, which violates the scale-freeness of the network. In a higher threshold value of 0.45 (Figure 2C), 4.8% of nodes, an average of 13 nodes per network are excluded from the largest component, which we considered inappropriate for a whole-brain analysis. Therefore, after searching the entire population of 180 subjects, the threshold value was determined to be 0.40 (Figure 2B). For the HCP dataset, the threshold value was set to 0.36, which resulted from a similar procedure.

### Embedding of the Graphs Into Spaces of Various Curvatures/Dimensions

The average distortion (D_avg_) and mean average precision (mAP) of embedding between the target manifolds from the HCP and ABIDE datasets are shown in Tables 1 and 2. In general, embedding into the spaces of various curvatures with the same dimension did not show any significant difference in terms of mean average precision and average. Embedding into the manifold with higher curvature showed better quality of embedding. In the embedding of hyperbolic manifolds, more cases have converged compared with Euclidean and spherical manifolds.

**Table 1.** Fidelity measures of embedding into various manifolds for the HCP dataset. For the HCP dataset, embedding into the spaces of various curvatures with the same dimension did not show any significant difference in terms of mean average precision and average. Embedding into manifold with higher curvature showed better quality of embedding. In the embedding of hyperbolic manifolds, more cases have converged compared with Euclidean and spherical manifolds. D_avg_ = average distortion. mAP = mean average precision.

Embedded space	Fidelity measures (Mean ± *SD*)		Converged case
	D_avg_	mAP	
10-dimensional spaces			
𝔼^10^	0.0833 ± 0.0104	0.9039 ± 0.0388	120 (66.7%)
ℍ^10^	0.1020 ± 0.0382	0.8798 ± 0.0561	173 (96.1%)
𝕊^10^	0.1011 ± 0.0630	0.8459 ± 0.0874	125 (69.4%)
2- and 1-dimensional spaces			
𝔼^2^	0.1193 ± 0.0138	0.7083 ± 0.1390	123 (68.3%)
ℍ^2^	0.1308 ± 0.0243	0.7295 ± 0.1233	173 (96.1%)
𝕊^2^	0.1337 ± 0.0640	0.6856 ± 0.1328	127 (70.6%)
𝕊^1^	0.1657 ± 0.0471	0.5583 ± 0.1390	126 (70.0%)

**Table 2.** Fidelity measures of embedding into various manifolds for the ABIDE dataset. Embedding into the spaces of various curvatures in the ABIDE dataset showed similar results, not differing significantly among the same dimensions of manifold with different curvature, in terms of mean average precision and average. Embedding into manifold with higher curvature showed better quality of embedding. In the embedding of hyperbolic manifolds, more cases have converged compared with Euclidean and spherical manifolds. D_avg_ = average distortion. mAP = mean average precision.

Embedded space	Fidelity measures (Mean ± *SD*)		Converged case
	D_avg_	mAP	
10-dimensional spaces			
𝔼^10^	0.1042 ± 0.0153	0.8812 ± 0.0455	162 (65.3%)
ℍ^10^	0.1248 ± 0.0308	0.8230 ± 0.0681	248 (100.0%)
𝕊^10^	0.1136 ± 0.0174	0.8191 ± 0.0573	190 (76.6%)
2- and 1-dimensional spaces			
𝔼^2^	0.1378 ± 0.0146	0.5787 ± 0.1338	198 (79.8%)
ℍ^2^	0.1507 ± 0.0180	0.5604 ± 0.1330	248 (100.0%)
𝕊^2^	0.1659 ± 0.0765	0.5673 ± 0.1245	183 (73.8%)
𝕊^1^	0.1810 ± 0.0144	0.4248 ± 0.1182	186 (75.0%)

### Embedding Graphs Into the Two-Dimensional Hyperbolic Disc

**Figure 3.**
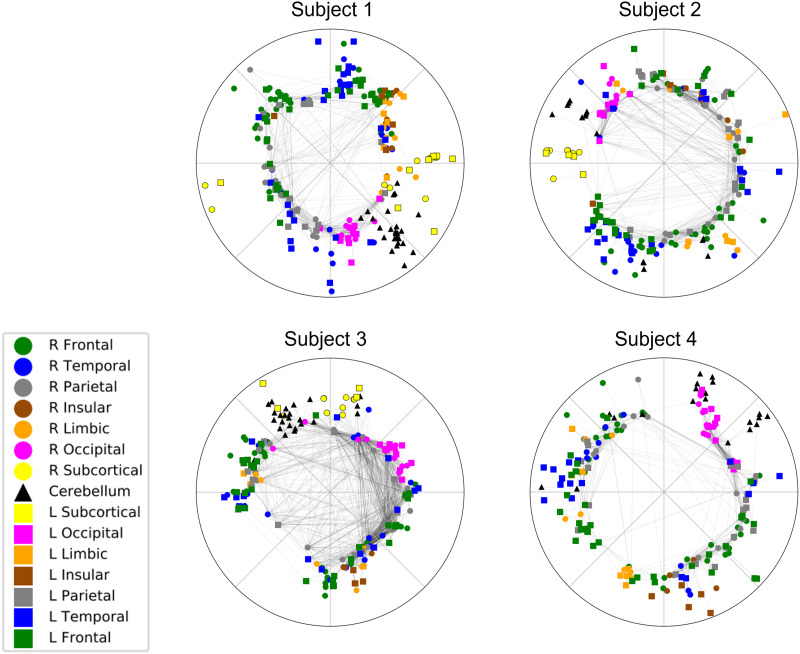
Embedding results from the 𝕊^1^/ℍ^2^ model. The figure demonstrates the results from four representative cases from the HCP dataset. Colors denote the anatomic lobe in which each ROI is located, while the lobes within the right, left cerebral hemisphere, and cerebellum are marked with circle, square, and triangular markers, respectively. The embedding result revealed vacant space in the center of the disc. ROIs from the bilateral insula, subcortical, and cerebellum had narrow distributions of angular dimensions. ROIs from larger anatomic lobes (frontal, temporal, parietal) were located with relatively broader distributions. ROIs from the same lobes of both hemispheres (for example, right and left frontal lobes) had similar distributions of angles.

Results of the embedding (in the ROI scope) of the representative cases onto the 𝕊^1^/ℍ^2^ model are shown in Figure 3. Colors denote the anatomic lobe, while the lobes within the right and left cerebral hemispheres and cerebellum are marked with circular, square, and triangular markers, respectively. The results of embedding in the voxel scale are shown in Supplementary Figure 2 (see the Supporting Information).

Every embedding result revealed vacant space in the center of the disc. ROIs from the bilateral insula, subcortical, and cerebellum had narrow angular distributions. ROIs from larger anatomic lobes (frontal, temporal, parietal) were located with relatively broader distributions. ROIs from the same lobes of contralateral sides (for example, right and left frontal lobes) had similar distributions of angles.

**Figure 4.**
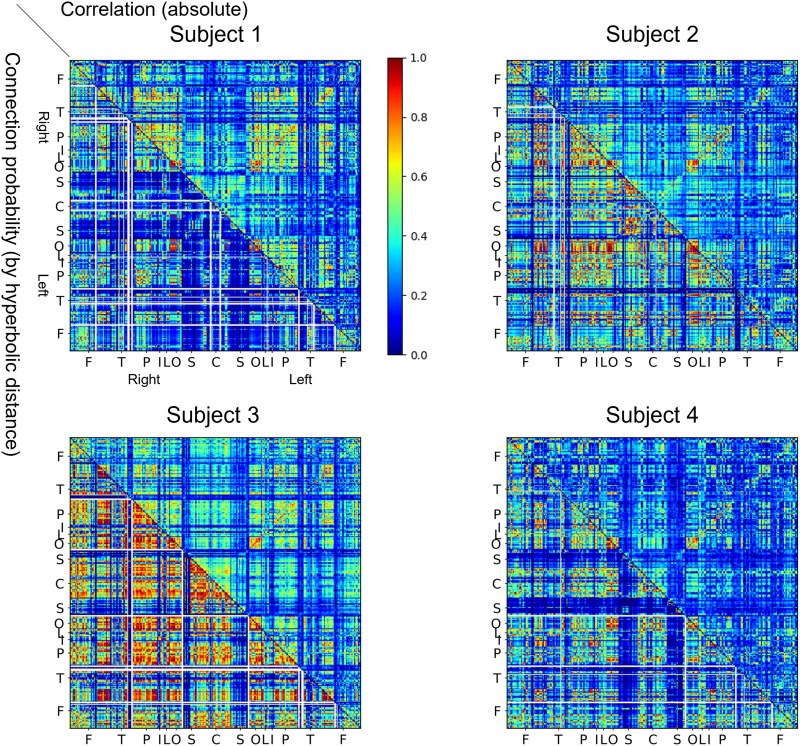
Connection probability versus absolute value of the correlation coefficient. The lower triangle demonstrates the connection probability calculated by *p*_*ij*_ = $\frac{1}{1+e^{\frac{\beta }{2}\left(d_{ij}-\hat{R}\right)}}$, where *d*_*ij*_ is the hyperbolic distance between nodes, β is the clustering coefficient, and $\hat{R}$ is the outermost radius of the node. The upper triangle denotes the absolute correlation value. The symbols in each axis denote the same anatomic lobe of the nodes as in Figure 1. The two measures tended to show similar patterns for each of the subjects (see Supplementary Note 4 in the Supporting Information).

**Figure 5.**
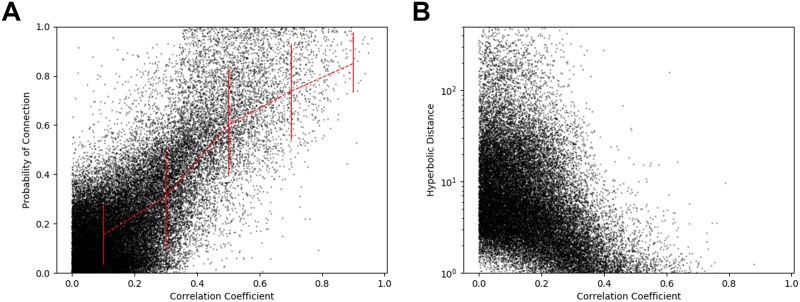
Correlation between proximity measures. Both figures show results for a representative subject. (A) The absolute value of the correlation coefficient and probability of connection between node pairs showed positive correlation for all of the subjects (*P* < 0.0001). The red line represents the mean value of the connection probability for each interval of the correlation coefficient and asymmetric error. (B) The same analysis using hyperbolic distance instead of probability of connection showed negative correlation for all of the subjects (*P* < 0.0001).

To assess the validity of embedding, we compared the absolute value of the original correlation coefficients used for thresholding with the connection probability between the edges, which resulted in hyperbolic distances computed on the 𝕊^1^/ℍ^2^ model (Figure 4). The two measures, which are commonly scaled from 0 to 1, were correlated positively for each of the subjects (*r* = 0.4176 ± 0.1494, *P* < 0.0001 for all subjects), while the hyperbolic distance was negatively correlated with the correlation coefficient between node pairs (*r* = −0.0551 ± 0.0304, *P* < 0.0001 for all subjects). Figure 5 demonstrates the scatter diagrams of the proximity measure for one representative subject. This demonstrates that the two proximity measures have similar patterns. (See Supplementary Note 4 in the Supporting Information.)

To investigate whether the embedding results are compatible with other established methods of functional segregation, we made use of network components calculated by ICA and plotted the composing voxels of each independent component in colors in the 𝕊^1^/ℍ^2^ disc (Supplementary Figure 3 in the Supporting Information). To remove the arbitrariness of rotation by the constant angle in the embedded discs, we adjusted the angular position of the embedded discs in order for the mean angular coordinate of nodes in the VN to have a value of 0 (Supplementary Figure 5 in the Supporting Information). However, rotational symmetry should be considered to see the distribution of each component and belonging voxels thereof.

We assessed the reproducibility of the embedding process by measuring the coefficient of variance for the hyperbolic distance matrices in one representative case (Supplementary Figures 4 and 6 in the Supporting Information). Most of the nodes had relatively low arbitrariness of position in the repeated embedding procedure. Meanwhile, outermost-positioned nodes, which represent low popularity, tended to have a position with higher variability (Supplementary Note 5 in the Supporting Information).

### Detection of Abnormal Functional Pathways on Hyperbolic Disc in the ASD Subjects

**Figure 6.**
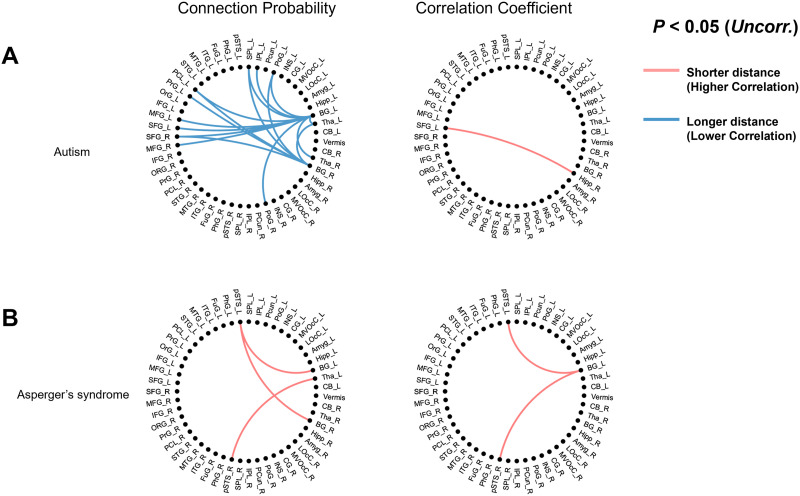
Detection of abnormal pathways in autism subjects. The 274 ROIs were reduced to 51 brain subregions for the sake of clarity of visualization. The lines in the circular diagrams represent the abnormal connection probability or correlation compared with the control group composed of 134 healthy adults (uncorrected *P* < 0.05). (A) One subject with autism showed longer hyperbolic distances of edges connecting frontoparietal cortices and bilateral basal ganglia, which represent the cortico-striatal pathway, compared with the normal group. The pattern was not clear in the means of the correlation coefficients. (B) Another subject with Asperger's syndrome showed shorter hyperbolic distances of edges that connect bilateral posterior superior temporal sulcus (pSTS) and basal ganglia. The correlation study showed a similar pattern. The list of abbreviations in the circular diagrams is noted in Supplementary Note 6 (see Supporting Information).

Among the variable patterns of functional brain networks embedded on the 𝕊^1^/ℍ^2^ model from the ASD subjects, one subject of autism spectrum disorder showed a longer hyperbolic distance of node pairs connecting the bilateral frontoparietal cortices and bilateral basal ganglia, which represent the cortico-striatal pathway, compared with the control group of 134 healthy young adults (Figure 6A; *P* < 0.05). This tendency was not prominent when the comparison analysis was conducted on correlation matrices of a subject and 134 controls.

Notably, a subject with Asperger's syndrome showed a shorter hyperbolic distance of the edges of node pairs, which connect bilateral posterior superior temporal sulcus (pSTS) and basal ganglia, and the correlation study showed a similar pattern (Figure 6B; *P* < 0.05).

## DISCUSSION

Neuroscientific work in the past decades has provided the perspective that the brain is a complex network that is composed of interactions between regions (Sporns, 2011), and rs-fMRI has emerged as an efficient tool for exploring the connection (van den Heuvel & Pol, 2010). However, it still remains unclear how each region of the brain is connected functionally in a multiplex way and organized in higher order to perform its complicated function at rest with activation whose amplitude was smaller than when related with tasks. Preceding work by Allard et al. (Allard & Serrano, 2020; Allard et al., 2017) implemented a hyperbolic geometry model to enlighten this question by providing a map of structural connectomes, while exploiting this geometry’s expansive space able to contain the brain connectome’s high-order and high-dimensional correlational structure. One of the major advantages of this model is that it implements two-dimensional hyperbolic space to embed the network, which makes it easy to visualize brain connectomes and to understand how the brain regions are working in a multiplex connected fashion.

Meanwhile, not much work has attempted to yield deep insight into the subtle correlative work of multiple nodes of an individual brain from the norms made of many subjects as represented by an individual functional map to define an unheralded disease-specific anomaly of a single affected subject. Since many neurologic disorders are classified as a group of similar clinical manifestations (Thapar et al., 2017) with unclear neurologic pathophysiology, changing the scope into connectomes of affected individuals might significantly help in further classifying the disease entities and provide information on the treatment of disease. Furthermore, this work inherits the advantage of the preceding work by Allard and Serrano (2020), that the resulting object is represented in two-dimensional space both for visualization and for further analysis to disclose which internodal connections deviate from the norms in these hyperbolic discs.

### Composition of the Network

To compose a framework of the functional brain network, we implemented interregional time series correlations of rs-fMRI, which grant a stable measure of the resting brain (Greicius et al., 2003). We applied the *absolute value* of Pearson's correlation between brain regions, which regards the two brain regions with anticorrelated time series as connected, as same as the correlation of the same strength. Anticorrelation between specific brain regions has a role in organizing functional brain architectures (De Havas et al., 2012; Keller et al., 2015; Kucyi et al., 2018). Then, we thresholded the matrix to obtain a binary graph for each network. In this study, we decided to apply the same threshold among patients in a dataset for comparability.

Since only the largest component can be embedded in the model, we needed to retain as many nodes as possible. If we apply the higher threshold value, we drop more nodes from the network, which makes it difficult to compare individuals and establish the difference that is clinically meaningful from the embedding of the whole-brain networks. On the other hand, if we apply a threshold value that is too low, the binary networks become denser with almost every edge connected, and the assumption of scale-freeness is violated as we perform maximum likelihood estimation for embedding the functional brain connectomes to the hyperbolic disc using the 𝕊^1^/ℍ^2^ model embedding. Thus, we composed the sparsest network while maintaining most of the brain regions in its largest component. We assumed that we could remove noise and/or artifacts in the observational data and also emphasized the multidimensional, tree-like, internodal correlational structure of the functional brain networks.

### Degree Distribution of the Network

In order to congruently embed the network in the hyperbolic disk onto the 𝕊^1^/ℍ^2^ model, it was believed that clean scale-freeness was a prerequisite of embedding (Boguñá et al., 2009; Krioukov et al., 2010). However, recent studies have uncovered that even in networks that have degree distributions other than strict power law, embedding with high fidelity can be performed (Allard & Serrano, 2020). However, in this work, we wanted the network to follow power law as much as possible, adopting a rather conservative viewpoint.

That a network is scale-free means that the structure of the network has a similar structure independent of the scale of observation (Barabási & Bonabeau, 2003; Voitalov et al., 2019). In detail, most nodes have a small number of connections, while some nodes have a large number of functional connections with other nodes. This indicates some of the topological features of the functional brain network, such as the hierarchical organization of nodes (Sporns et al., 2004), as its structural counterpart (Faskowitz et al., 2018). Heterogeneity of degree distribution implies various models, including hyperbolic, and we adopted a hyperbolic model in this investigation.

### The Geometry of the Brain Network

Real-world networks can be embedded in a physical space corresponding to their structure and organization (Chami et al., 2019; Smith et al., 2019). In this study, the architecture of the high-order, high-dimensional, internodal correlational structure of the functional brain network, of which a structural counterpart lies in three-dimensional Euclidean space, was investigated by comparing and computing the fidelity measures of embedding in manifolds of positive (spherical), negative (hyperbolic), and zero (Euclidean) curvatures. Our result showed no significant difference in the quality of embedding in hyperbolic or spherical spaces compared with the Euclidean spaces, which indicates that embedding into hyperbolic space is as good as representing the network in Euclidean or spherical space. Fidelity of embedding showed trends of a better result as the manifolds got to a higher dimension, but here we stick to a two-dimensional disc because of its feasibility of visualization.

Space of hyperbolic geometry is, in fact, the place we live in, as indicated by the special relativity with multilayered, hierarchical, and multiplex-correlated way. The most characteristic feature of the hyperbolic space compared with other types of space is that it best represents the data with hierarchical structure (Lazcano et al., 2021), as in deep neural networks implemented in deep learning (Peng et al., 2021). Prior research about embedding into non-Euclidean spaces also reports the hyperbolic space as the matching geometry to embed tree-like structures, employing the least distortion (Gu et al., 2018; Sala et al., 2018). This reveals the tree-like nature of the network, following from the nature of the hyperbolic space that the geodesic passes a path near the origin, as the shortest path in the tree graph passes their common parent node. Note that functional brain networks form a tree structure, not in a three-dimensional space of real life, but in a higher dimensional space derived from the correlations of nodes during 5 min (ABIDE) or 15 min (HCP) of rs-fMRI acquisition based on the stationarity assumption. The hyperbolic geometry explains that the graphs derived from these internodal functional brain connectomes that represent the brain networks exhibit a hierarchical and tree-like organization, which is suggested in prior research of humans or other animals (Bardella et al., 2016; Telesford et al., 2011).

### Hyperbolic Disc Representation of the Network

Since it has been known that many real-world networks have hyperbolic properties, many investigators have proposed algorithms for figuring out how to generate geometric objects that are most likely to generate the given network (Keller-Ressel & Nargang, 2020; Muscoloni & Cannistraci, 2018; Papadopoulos et al., 2014; Suzuki et al., 2019). The present study makes use of Mercator (García-Pérez et al., 2019), using Laplace eigenmaps for the reduction of dimension and maximum likelihood estimation techniques for acquiring the most appropriate geometric object representing the original network (Papadopoulos et al., 2014). By these means, we could visualize the functional brain network in the hyperbolic disc with the conformal structural properties with the real-world network (Papadopoulos et al., 2015; Papadopoulos et al., 2012). Note that the two embeddings we used for testing fidelity of embedding on the spaces with various curvatures/dimensions and for final hyperbolic disc embedding represent two different procedures. In the former task for testing embedding spaces, we used the learning procedure by minimizing the loss function (Gu et al., 2018), while in the latter task of the 𝕊^1^/ℍ^2^ model embedding, we used Mercator (García-Pérez et al., 2019) for reduction of dimension and maximizing likelihood for the geometric object to generate the original binary graph (Papadopoulos et al., 2012).

The embedding procedure of the binary graph onto the hyperbolic disc according to the 𝕊^1^/ℍ^2^ model provides the hyperbolic distance as a measure of how the two brain nodes are functionally similar, that is, similar correlational structures derived from time series of node pairs. As an effective distance (Allard & Serrano, 2020), this combines factors that determine the connection probability between nodes (which are related as in Equation 6 in the Methods section). As a result, this procedure effectively visualizes the network to give a quantitative comparison of the functional brain network among individuals.

The geometric meaning of the 𝕊^1^/ℍ^2^ model is that the popularity and similarity of the nodes are represented by the radial and angular coordinate of the two-dimensional hyperbolic space, respectively (Papadopoulos et al., 2012). The basic assumption is that the closer nodes are more likely to be connected. In other words, the short hyperbolic distance between the two nodes correlates with a high probability of connection in the generated network, and this process of embedding helps us to understand the topology of the network (Alanis-Lobato et al., 2016).

The radial representation of the embedded nodes of the network represents hub characteristics or popularity. That is, nodes with higher degrees are located closer to the center of the disc. From this perspective, we point out that in all of our embedding results, centers of embedded discs were vacant. This implies that the functional brain network does not have any nodes with dominant high popularity, implying that functional brain networks have a decentralized information flow.

Previous similar studies performed with other kinds of real networks showed variable patterns. The network of the World Trade Atlas in 2013 (García-Pérez et al., 2016) showed two global hubs remarkably close to the center of the disc, named “USA” and “China.” These two nodes work as the global hub, which generally has a high connection probability with any other nodes from the whole network. On the other hand, the embedded map of internet connection (Boguñá et al., 2010) did not show heavily centered nodes, but several locally centered nodes were seen, which might function as a local hub of the system. Finally, the map of the structural brain network (Allard & Serrano, 2020) showed a relatively uniform distribution of radial coordinates without a noticeably centered node.

The mapping result of the functional brain network is most similar to the result from the structural network, which indicates that there is no definite global hub connected with the vast substructures of the network. This might result from the fact that both structural and functional brain networks are based on the real neuronal network of the brain, of which the number of connections (i.e., degree of the node) is limited by physical constraints. Note that in the former two examples, the number of connections for each node is not physically limited. This does not mean that the hierarchy does not exist: The nodes have variation in their radial coordinates, which means it does exist in the functional brain network.

From the tendency that nodes from the same anatomic lobe tend to cluster in a similar section on the angular coordinates, we could address a certain degree of neuroanatomical relevance, which is reported from previous literature on functional brain networks (Power et al., 2011). The regions involved in sensory or motor function such as subcortical regions, occipital lobes, and cerebellum showed relatively strong concentration in narrow sections of angular coordinates, while the association cortices such as frontal, temporal, and parietal regions were broadly distributed in angle.

Another notable feature is that nodes from the same lobes of both hemispheres, which are also denoted as the homotopic area, tend to cluster in similar angular coordinates, wherever they are located. This is markedly different from the map of the structural brain network, in which the different hemispheres showed separation on angular coordinates. As homotopic connection in the brain is reported by prior research (Lee et al., 2008; Mancuso et al., 2019; Wei et al., 2017), our results suggest a stronger homotopic connection in functional connectivity, more than indicated by structural connection.

Note that the set of positions of nodes has symmetry upon reflection and rotation (Ganea et al., 2018) and branch permutation invariance (Alvarez-Melis et al., 2020), which inevitably arise when we adopt hyperbolic embedding of any kind, Poincare or other hyperbolic ones. These are key features to consider when one compares a (group of) disc to another, and implementing the hyperbolic distance between two nodes, which is a *relative* measure, resolves the reflection and rotational symmetry issue. Since hyperbolic distance is an essential measure of this 𝕊^1^/ℍ^2^ model because it is the single argument that determines connection probability between two nodes of the disc, we could also assure the reproducibility of the result in terms of hyperbolic distance.

### Abnormality of Functional Pathways in the Diseased Subjects

The autism spectrum disorder is a diagnostic group of neurodevelopmental disorders with deficits in communication and social interaction and repetitive, restrictive behaviors (Baio et al., 2018). A vast number of studies in neuroscience have accumulated sufficient data to make clear that ASD is a disorder of abnormal connectivity of neural pathways (Belmonte et al., 2004; Bullmore & Sporns, 2012; Just et al., 2004; Maximo et al., 2014), in spite of its complex and heterogeneous nature (Kleinhans et al., 2008).

We assessed the clinical applicability of our method by detecting abnormalities of the network in ASD subjects compared with the control group. While the abnormality of the network on hyperbolic discs among subjects was variable, some subjects showed results congruent with clinical context and literature reports. The striatum is pointed to as the key of the pathophysiology of autism (Green et al., 2016; Uddin et al., 2013), and one of the subjects showed abnormal hyperbolic distance in pathways connecting the frontoparietal cerebral cortex and striatum (Figure 6A).

Asperger's syndrome, a high-functioning form of autism, is characterized by a higher intelligence and better-than-average verbal skills, but impaired nonverbal communication and social interaction, with restrictive and repetitive behaviors as in classic autistic patients (Frith & Mira, 1992; Wing, 1981). Though our study has presented the case of a single subject with Asperger's syndrome, the results showed an abnormal connection pattern of edges connecting the bilateral posterior superior temporal sulcus (pSTS) and subcortical regions, compatible with the literature (Isik et al., 2017; Materna et al., 2008). The bilateral pSTS is known to be associated with social interactions, and some prior literature reports its relevance with ASD. Our results suggest the abnormal connection of pSTS, which is consistent with impairment of social interaction in high-functioning autism patients, while the cortico-striatal pathway was relatively intact.

There are several limitations of this analysis. First, in the composition of the network, we used the absolute value of the correlation coefficient, which regards the anticorrelation between brain regions the same as correlation of the same absolute value. Since the anticorrelation between brain regions is believed to have a distinct nature (Fox et al., 2009; Liang et al., 2012), we should consider multigraphs to take both types of correlation into account in a future study. Second, the threshold we adapted for composition of the network was a fixed value for all subjects; this approach could be improved by adapting an optimal threshold in each individual subject. In this point of the study, we adapted a fixed threshold value for reproducibility, after qualitatively checking the degree distribution for individual subjects. Third, the embedding we performed in searching for the best fit manifold among spaces with various curvatures/dimensions is not the same as we have done finally to represent functional brain networks on hyperbolic discs according to the 𝕊^1^/ℍ^2^ model, and accordingly, findings from the former investigation of comparison between various space embeddings might not guarantee the highest quality of finally used hyperbolic disc embedding over any other choices. Last, we did not perform the multiple comparison adjustment in detecting the abnormality of the network, which reduces the statistical significance.

In conclusion, in this work, we aimed to find the most appropriate geometry for functional brain networks based on interregional time series correlation of rs-fMRI, and we embedded the networks onto two-dimensional hyperbolic discs of the 𝕊^1^/ℍ^2^ model. The results demonstrated the absence of global hub, homotopic functional coherence and a certain degree of functional-anatomical relevance, and yielded a reliable parameter of hyperbolic distances on this hyperbolic disc. When this method was applied to the diseased subjects, we detected abnormal pathways by comparing internodal hyperbolic distances. This leads us to a new method of reproducible visualization of functional brain networks with high fidelity from correlational functional brain networks and anomaly detection of internodal pathways in the brain networks of diseased subjects.

## ACKNOWLEDGMENTS

We deeply appreciate Dr. M. Ángeles Serrano (Universitat de Barcelona) for helpful advice on the methodology and Dr. Seonhee Lim (Seoul National University) for her help and essential advice in constructing the theoretical basis of the research and further comments that greatly improved the manuscript.

## SUPPORTING INFORMATION

Supporting information for this article is available at https://doi.org/10.1162/netn_a_00243.

## AUTHOR CONTRIBUTIONS

Wonseok Whi: Data curation; Formal analysis; Investigation; Methodology; Software; Visualization; Writing – original draft. Seunggyun Ha: Conceptualization; Data curation; Investigation; Methodology; Resources; Validation; Writing – review & editing. Hyejin Kang: Conceptualization; Formal analysis; Methodology; Project administration; Resources; Supervision; Validation; Writing – review & editing. Dong Soo Lee: Conceptualization; Formal analysis; Funding acquisition; Investigation; Project administration; Validation; Writing – review & editing.

## FUNDING INFORMATION

Dong Soo Lee, National Research Foundation of Korea (https://dx.doi.org/10.13039/501100003725), Award ID: 2017R1A5A1015626. Dong Soo Lee, National Research Foundation of Korea (https://dx.doi.org/10.13039/501100003725), Award ID: 2017M3C7A1048079. Dong Soo Lee, National Research Foundation of Korea (https://dx.doi.org/10.13039/501100003725), Award ID: 2020R1A2C2101069. Dong Soo Lee, National Research Foundation of Korea (https://dx.doi.org/10.13039/501100003725), Award ID: 2017R1D1A1B03032037.

## TECHNICAL TERMS

Fidelity of embedding: A measure that describes how the result of embedding preserves the features of the original graph (such as edge length or rank of edge length).
Tree-like graph: Connected graph whose structure is similar to a tree graph, but with a few short cycles.
Distance matrix: A symmetric square matrix that contains the pairwise distance between the elements of a set.
Autism spectrum disorder (ASD): A group of neurodevelopmental disorders that cause problems in social interactions and communication.
Clustering coefficient: A measure that describes how nodes in a graph are likely to cluster together.
Independent component analysis (ICA): An analytic technique that separates the observed multivariate data into statistically independent components.
Coefficient of variance (CV): A measure that describes the ratio of variability (standard deviation) to the mean of a variable.
Asperger’s syndrome: A neurodevelopmental disorder, which is a high-functioning subtype of ASD, with impaired nonverbal communication and social interactions.
Maximum likelihood estimation: A technique that determines parameters of a model that maximize the given likelihood function.
Laplace eigenmaps: A linear projection of a higher dimensional object onto a lower dimensional space, which preserves distances.
Homotopic area: Mirror area of the brain hemisphere.
