# Supplementary material for: Hyperbolic disc embedding of functional human brain connectomes using resting-state fMRI
Source: Netw Neurosci. 2022 Jul 1;6(3):745–64. doi: 10.1162/netn_a_00243 (PMC9810369; doi:10.1162/netn_a_00243)
Supplement: Supplementary file 1 [file netn-06-745-s001.pdf]

Whi, W., Ha., S., Kang, H., & Lee, D. S. (2022). Supporting information for “Hyperbolic disc embedding of functional human brain connectomes using resting-state fMRI.” *Network Neuroscience*, *6*(3), 745–764. https://doi.org/10.1162/netn_a_00243

Supplementary Information for

Hyperbolic disc embedding of functional human brain connectomes using resting state fMRI

Wonseok Whi[1,2], Seunggyun Ha[3], Hyejin Kang[4*], Dong Soo Lee[1,2,5*]

[1]Department of Molecular Medicine and Biopharmaceutical Sciences, Seoul National University, Seoul, Korea

[2]Department of Nuclear Medicine, Seoul National University and Seoul National University Hospital, Seoul, Korea

[3]Division of Nuclear Medicine, Department of Radiology, Seoul St. Mary's Hospital, The Catholic University of Korea, Seoul, Korea

[4]Biomedical Research Institute, Seoul National University Hospital, Seoul, Korea

[5]Medical Research Center, Seoul National University, Seoul, Korea

[*] Correspondence to H. K. (hkang211@snu.ac.kr) or D.S.L. (dsl@snu.ac.kr)

Contents

Supplementary Fig. 1 Windows of thresholds used for the subjects

Supplementary Fig. 2 Voxel-wise embedding onto hyperbolic discs by $\mathbb{S}^1/\mathbb{H}^2$ model

Supplementary Fig. 3 Plotting of independent components (ICs) using colors upon the voxel-wise hyperbolic embedded discs

Supplementary Fig. 4 Reproducibility of results (Repetition of the embedding)

Supplementary Fig. 5 Comparison of embedded hyperbolic discs of voxel correlational structures per each independent component (IC) over 10 subjects

Supplementary Fig. 6 Mean, standard deviation (SD), coefficient of variation (CV) of hyperbolic distance analog of a representative case, which were derived from 100 times-repeated hyperbolic embedding on $\mathbb{S}^1/\mathbb{H}^2$ model

Supplementary Note 1 Brief description of the geometric $\mathbb{S}^1/\mathbb{H}^2$ model derived from Serrano et al.[1] and Krioukov et al.[2]

Supplementary Note 2 Robustness of result, over different choices of threshold

Supplementary Note 3 Robustness of result, considering only positively correlated edges

Supplementary Note 4 Comparison of pattern of distribution between absolute correlation (derived from time-series correlation) and connection probability (after hyperbolic embedding and distance calculation between node pairs)

Supplementary Note 5 Coefficient of variation (CV) of repeated hyperbolic embedding and the hyperbolic distance analog between node pairs on this embedded hyperbolic disc

Supplementary Note 6 List of abbreviations in Fig. 7

References

Supplementary Figures

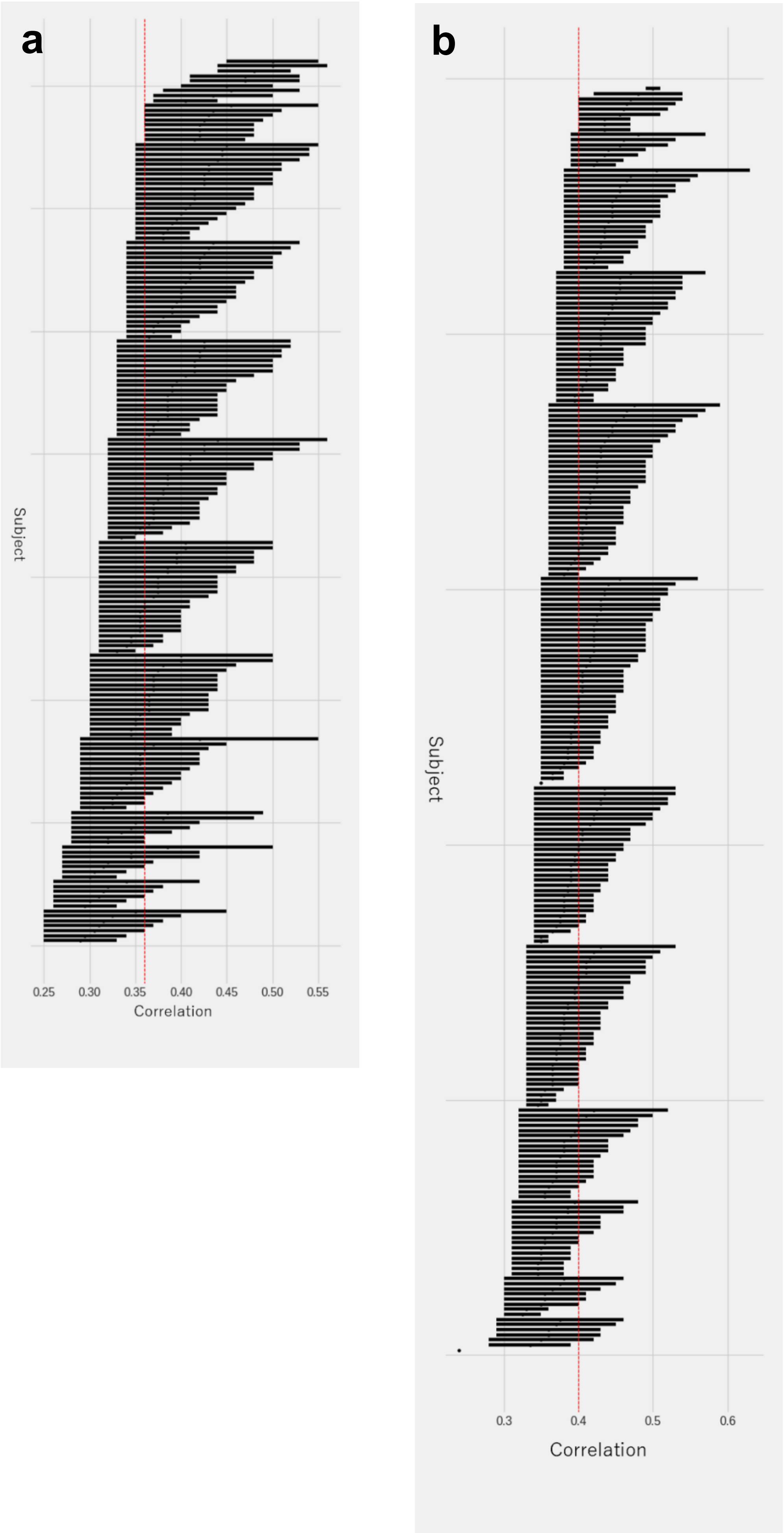
a
Subject
0.25 0.30 0.35 0.40 0.45 0.50 0.55
Correlation
b
Subject
0.3 0.4 0.5 0.6
Correlation

Supplementary Fig. 1 Windows of thresholds used for the subjects**.** (a) HCP dataset. (b) ABIDE dataset. The order of subject is sorted by lower limit of threshold, then by higher limit. We determined single fixed threshold value for each group by considering two factors, by reviewing networks with threshold of 0.15 to 0.65, with intervals of 0.01: a) degree distribution of network, determined by researchers if it follows power-law degree distribution. b) We wanted the network includes at least 95% (261 nodes, in case of 274-node networks in this work) of nodes of the original network.

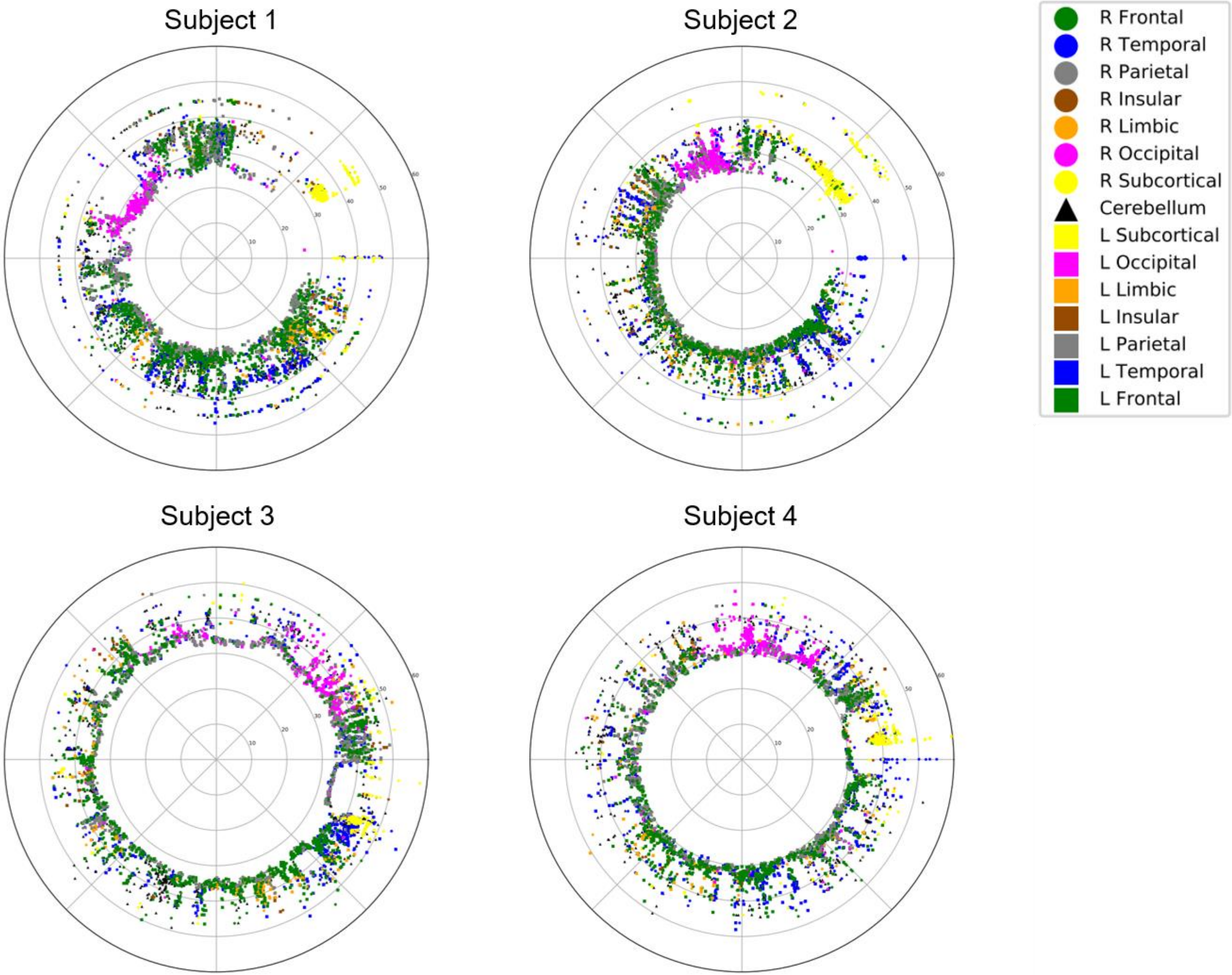
Subject 1
Subject 2
Subject 3
Subject 4
R Frontal
R Temporal
R Parietal
R Insular
R Limbic
R Occipital
R Subcortical
Cerebellum
L Subcortical
L Occipital
L Limbic
L Insular
L Parietal
L Temporal
L Frontal

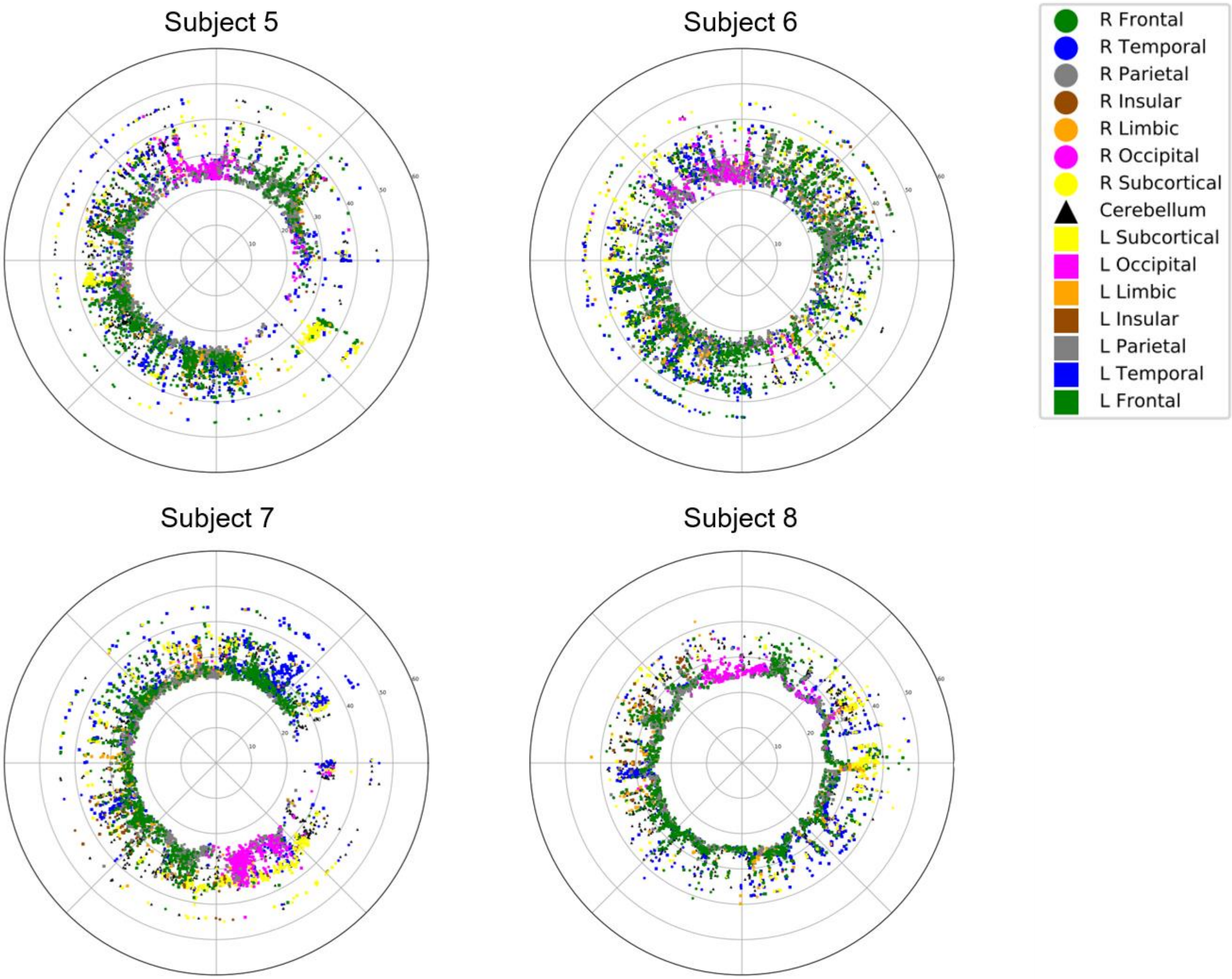

Subject 5
Subject 6
Subject 7
Subject 8
R Frontal
R Temporal
R Parietal
R Insular
R Limbic
R Occipital
R Subcortical
Cerebellum
L Subcortical
L Occipital
L Limbic
L Insular
L Parietal
L Temporal
L Frontal

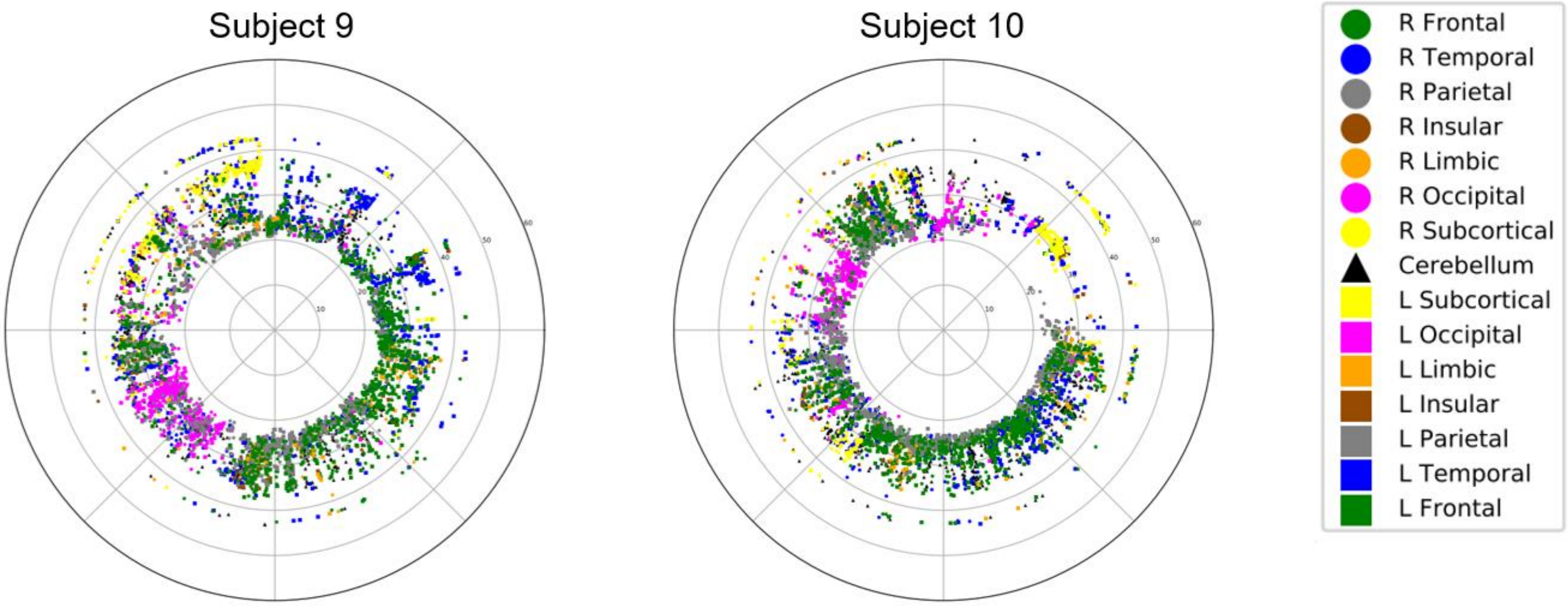

Supplementary Fig. 2 Voxel-wise embedding onto hyperbolic discs by $\mathbb{S}^1/\mathbb{H}^2$ model. Color denotes the anatomic lobe in which each region of interest (ROI) is located, while the lobes within the right, left cerebral hemisphere, and cerebellum are marked with circle, square and triangular markers, respectively. Similar to the result in ROI-scope, vacant space in the center of disc was prominent. Nodes in the same anatomic lobe tended to distribute in similar angular coordinate.

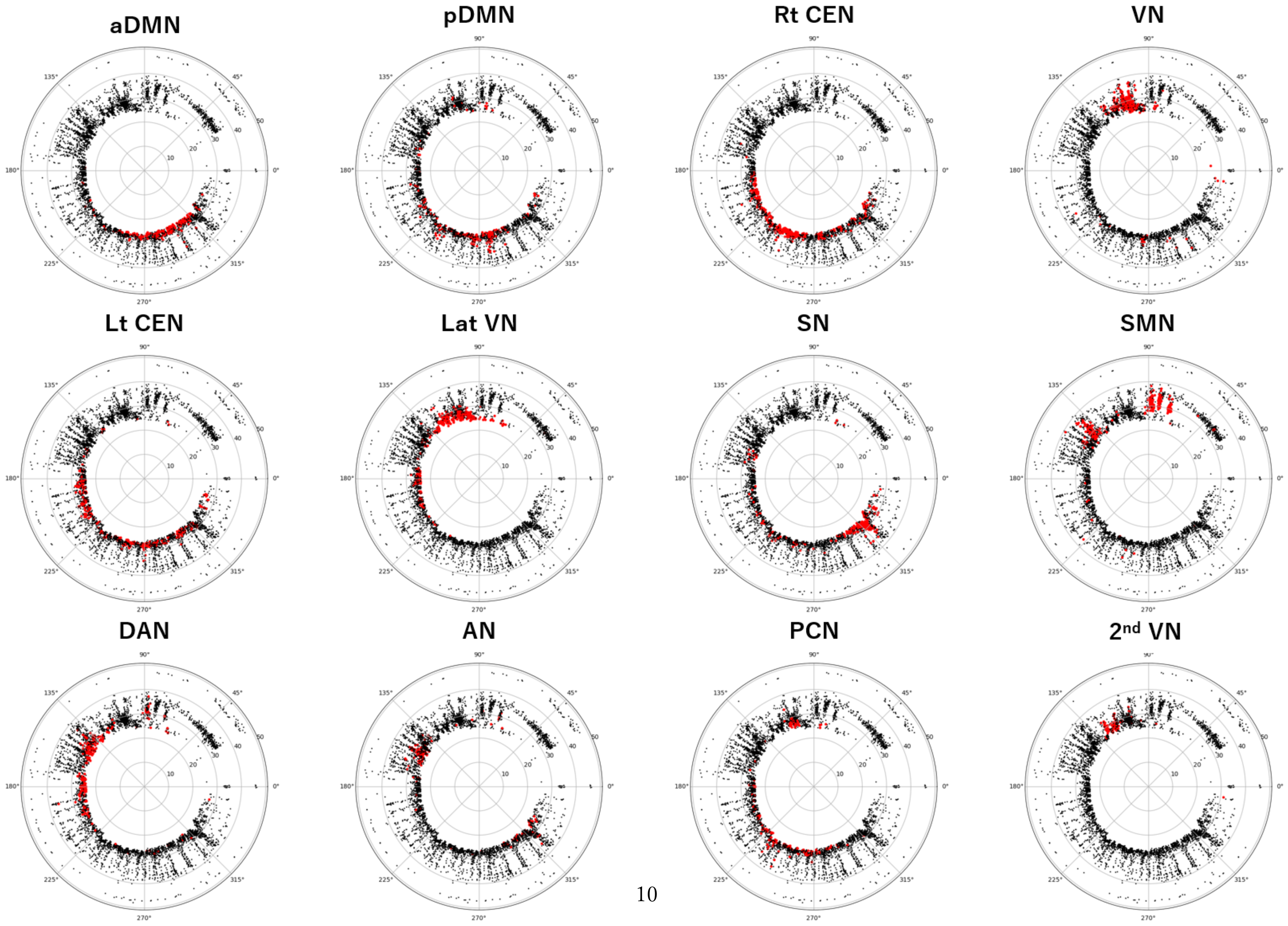
aDMN
pDMN
Rt CEN
VN
Lt CEN
Lat VN
SN
SMN
DAN
AN
PCN
2nd VN

Supplementary Fig. 3 Plotting of independent components (ICs) using colors upon the voxel-wise hyperbolic embedded discs. Using voxel data of HCP database, group-wise independent component analysis (ICA) was performed to yield 12 independent components (ICs). Voxel-wise embedded hyperbolic discs from 10 individual subjects were plotted with these ICs in color on the hyperbolic discs. A single representative case was used for visualization for this Figure. Voxels colored in red indicate the voxels included in each independent component (IC). aDMN = anterior default mode network, pDMN = posterior default mode network, CEN = central executive network, VN = visual network, Lat VN = lateral visual network, 2nd VN = secondary visual network, SN = salience network, SMN = sensorimotor network, DAN = dorsal attention network, AN = auditory network, PCN = precuneus network.

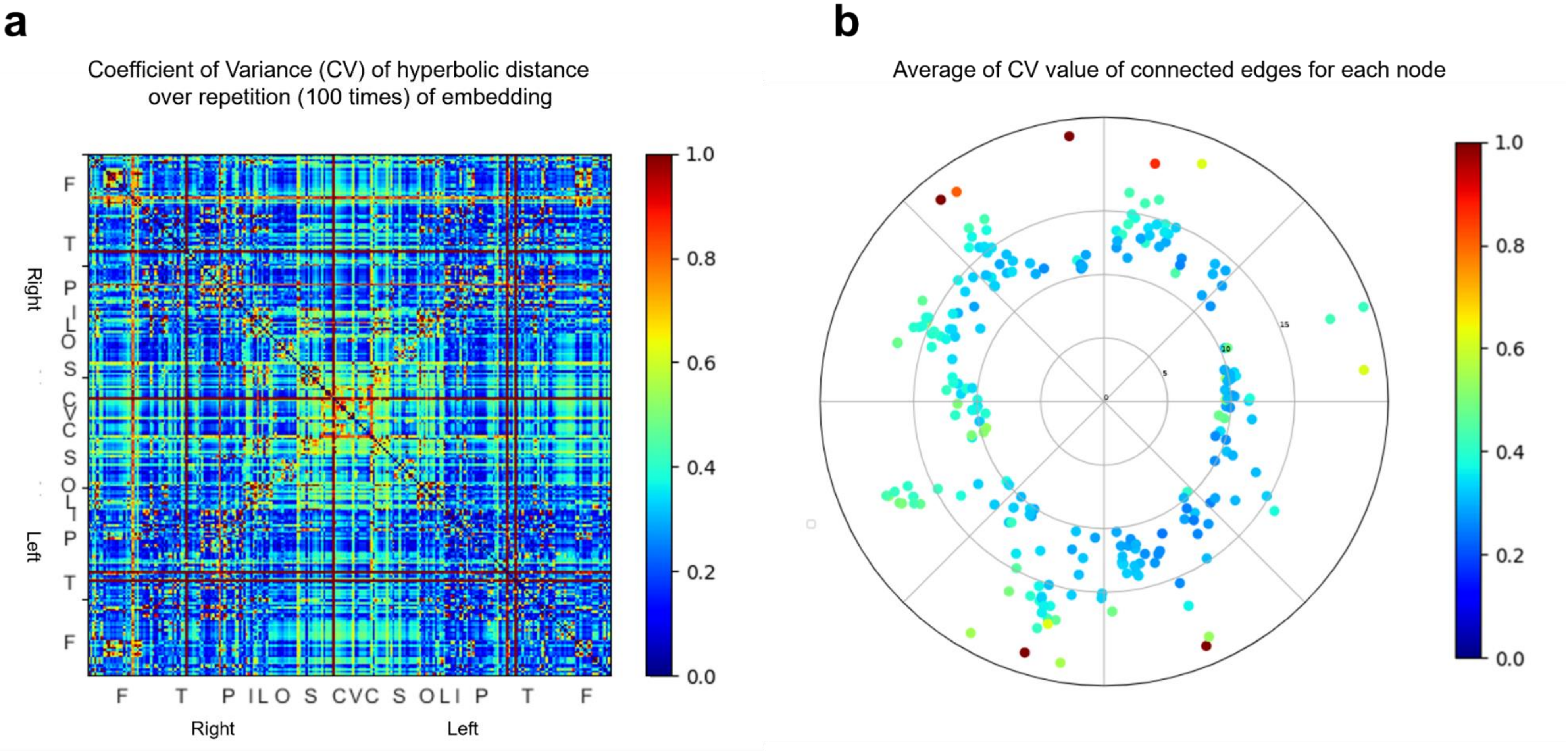

Supplementary Fig. 4 Reproducibility of results (Repetition of the embedding). We repeatedly performed the embedding procedure 100 times, in one

representative case. a Coefficient of variance (CV) of hyperbolic distances over repeated embedding (100 times) for each edge. The horizontal and vertical axis indicates the indices of vertices embedded in the $\mathbb{S}^1/\mathbb{H}^2$ model, which is ordered by lobes. The symbols in each axis denote the same anatomic lobe of the nodes as in Fig. 1. b Average of CV value plotted in a, calculated for each node over all connected edges with it. Blue nodes (low CV) represent nodes with a lower variance of position over embedding. Red nodes (high CV) represent a higher variance. The positions of vertices are positioned based on one sample result of embedding. Most of the nodes have relatively low arbitrariness of position in repeated embedding procedure. In contrast, outermost positioned nodes, which means nodes with low popularity, tended to have a position with higher variability.

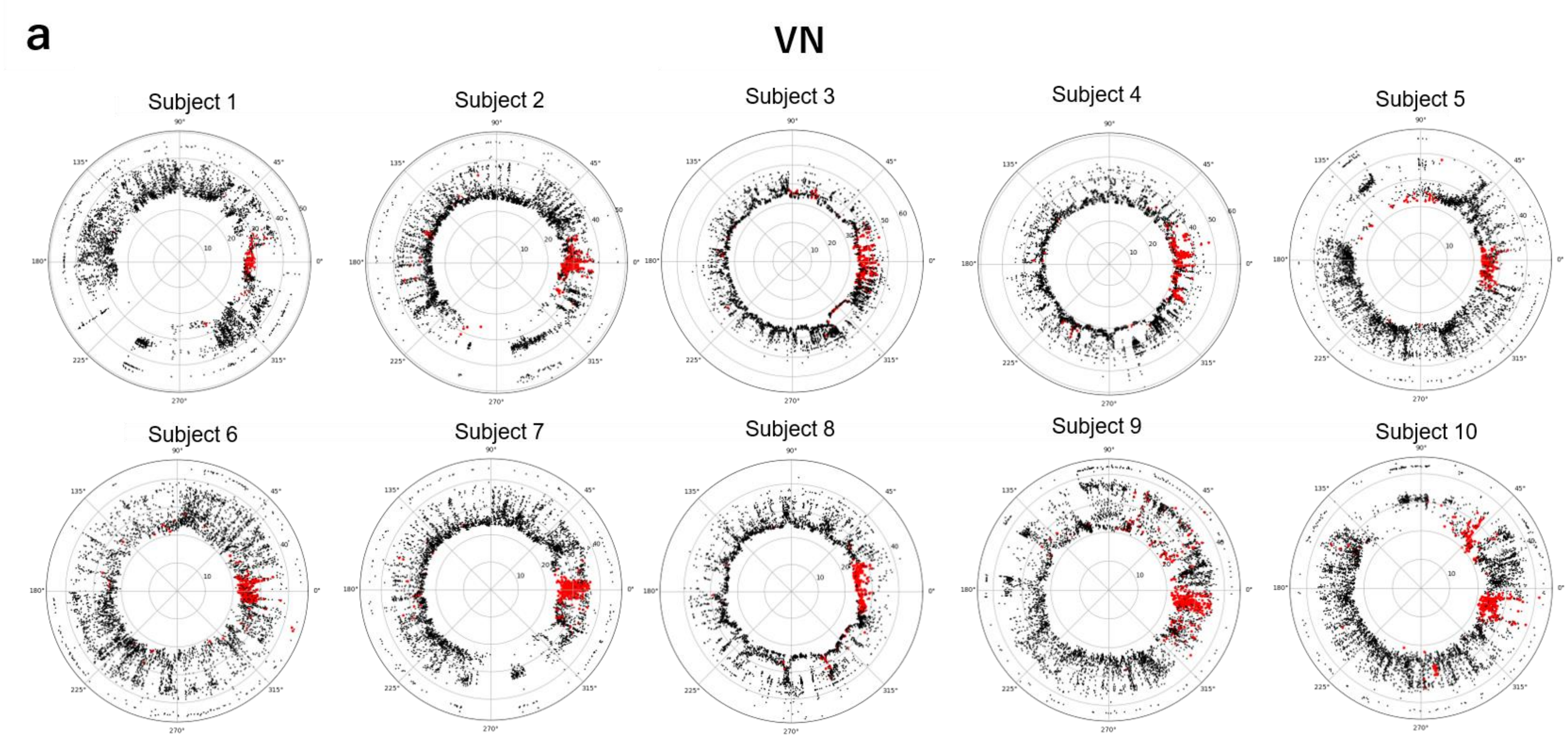
a
VN
Subject 1
Subject 2
Subject 3
Subject 4
Subject 5
Subject 6
Subject 7
Subject 8
Subject 9
Subject 10

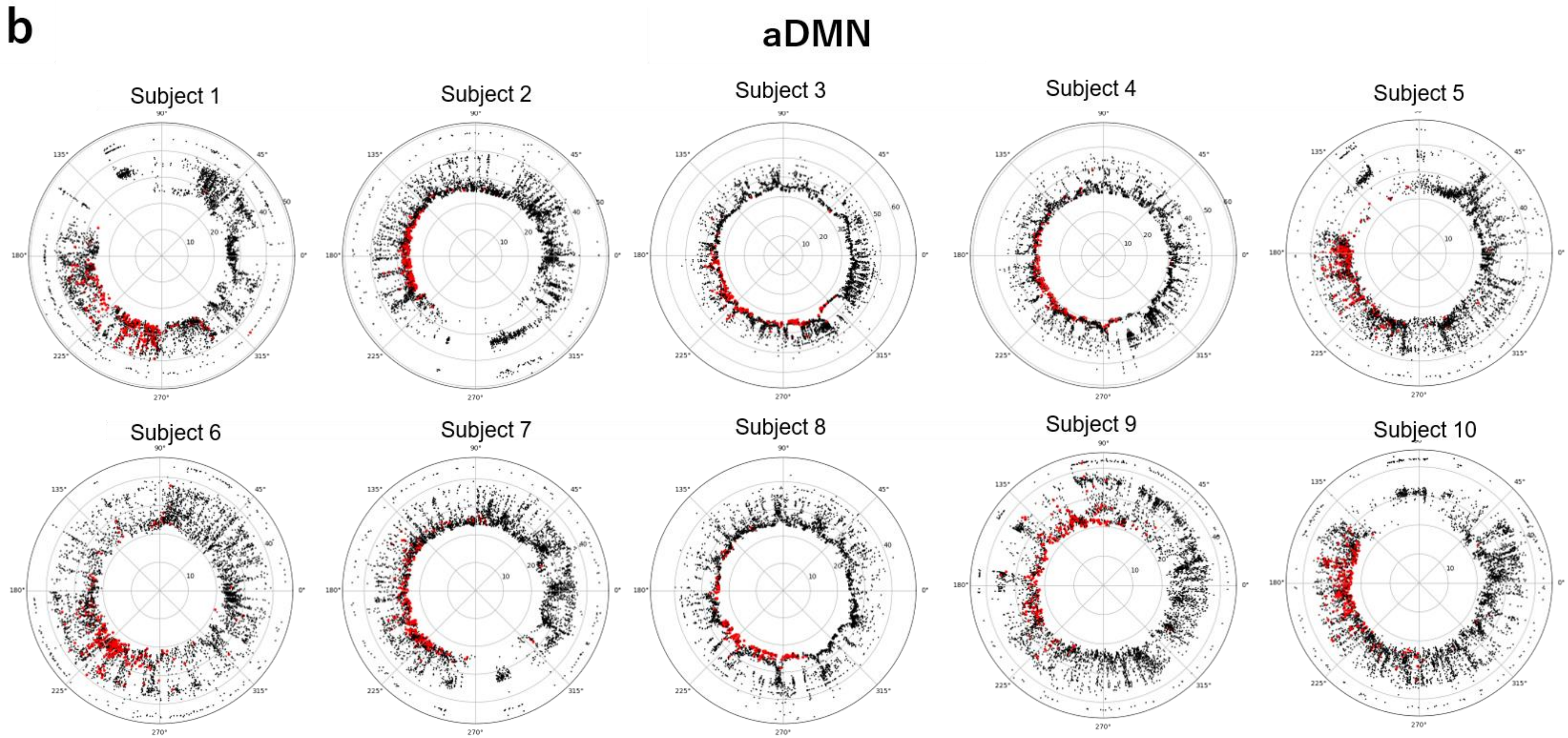
b
aDMN
Subject 1
Subject 2
Subject 3
Subject 4
Subject 5
Subject 6
Subject 7
Subject 8
Subject 9
Subject 10

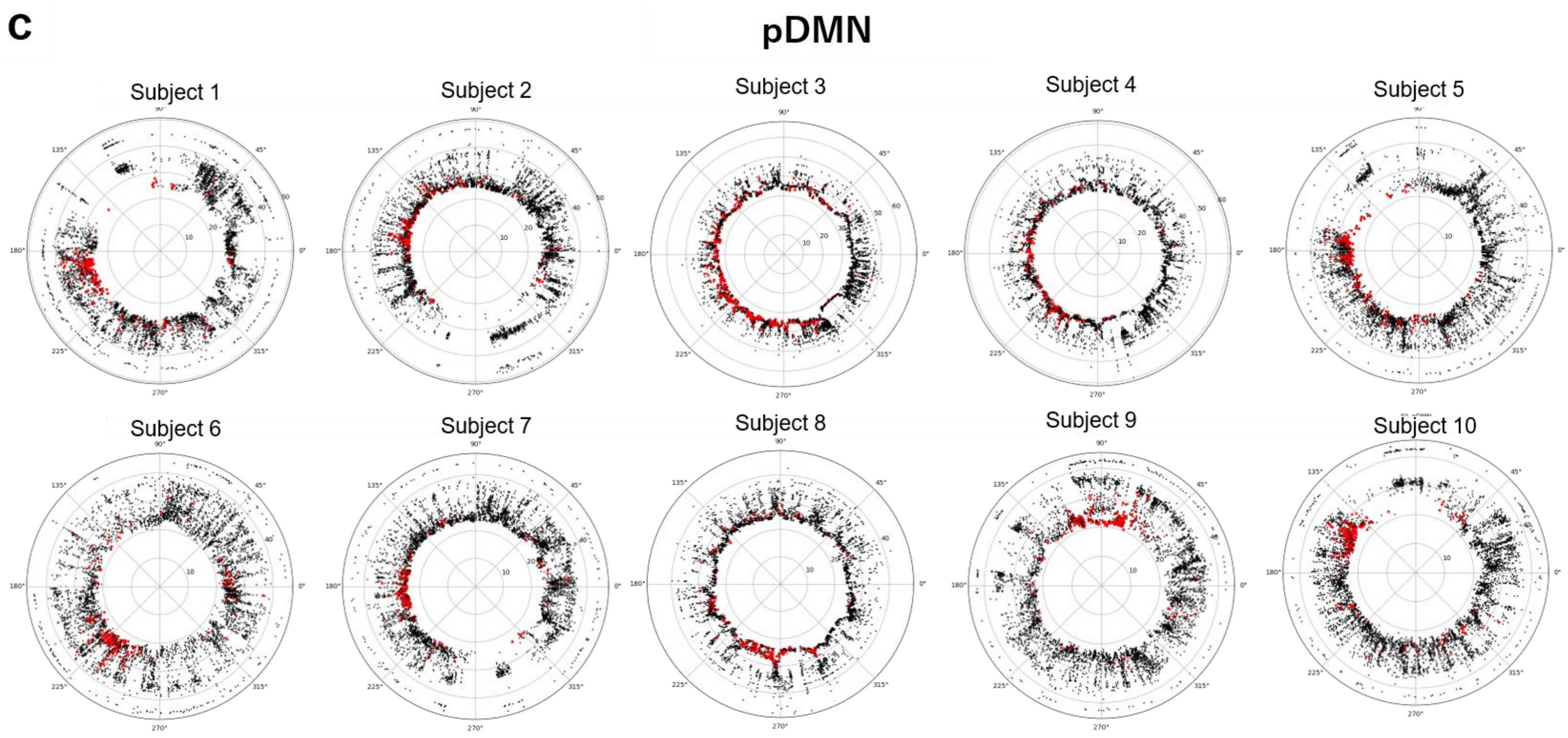
c
pDMN
Subject 1
Subject 2
Subject 3
Subject 4
Subject 5
Subject 6
Subject 7
Subject 8
Subject 9
Subject 10

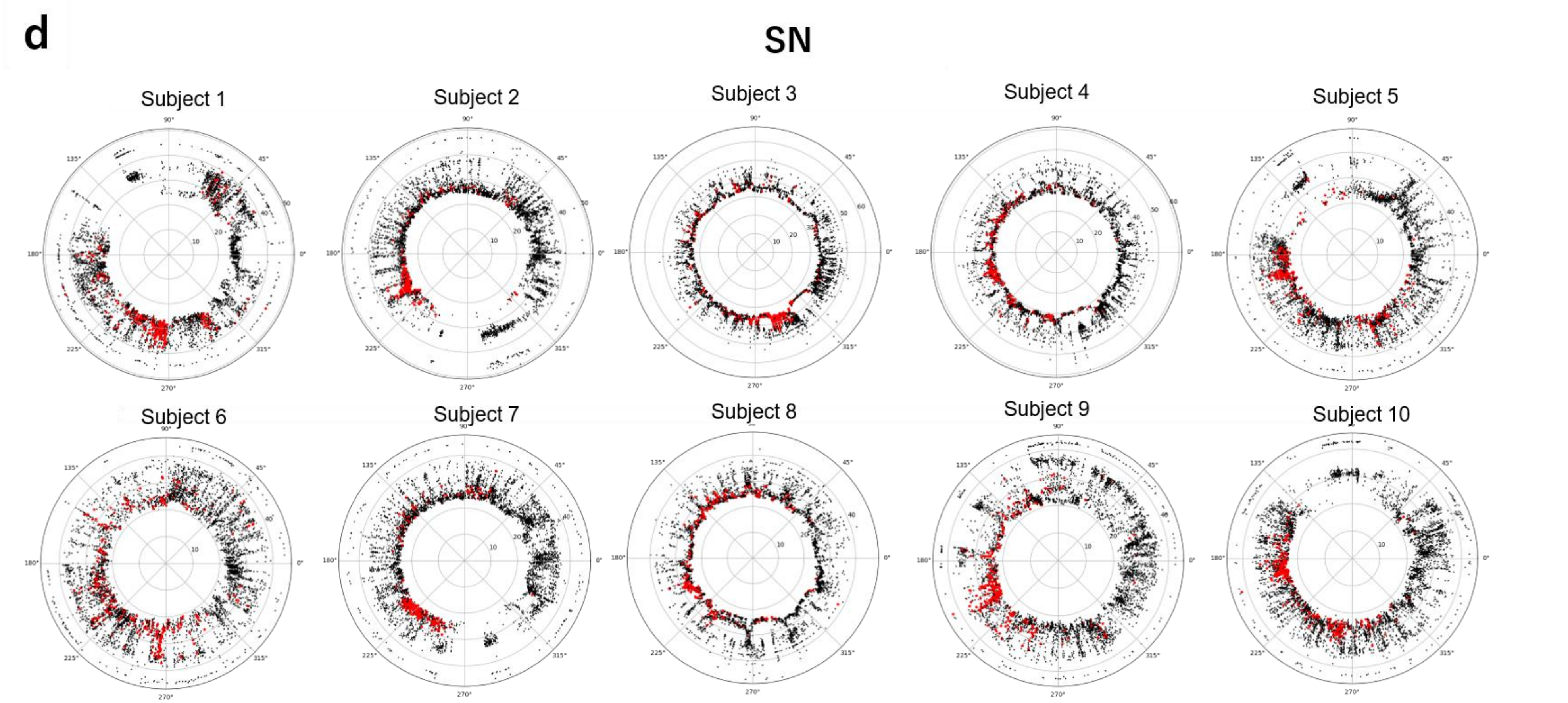

Supplementary Fig. 5 Comparison of embedded hyperbolic discs of voxel correlational structures per each independent components (ICs) over 10 subjects. Absolute angular position of the mean angle of visual network (VN) was set arbitrarily to have a value of angle 0 (right direction of the figure) on the

embedded hyperbolic disc. Thus, VN was located in the same angle, however, the angular coherence seemed to be higher for this IC VN. Colored independent components such as anterior DMN and posterior DMN in each subject showed ‘grossly similar but different in detail’ pattern of wider distribution than VN along individuals. IC of SN showed a broader distribution along the 10 individuals. IC: independent component, VN: visual network, DMN: default mode network, SN: salience network.

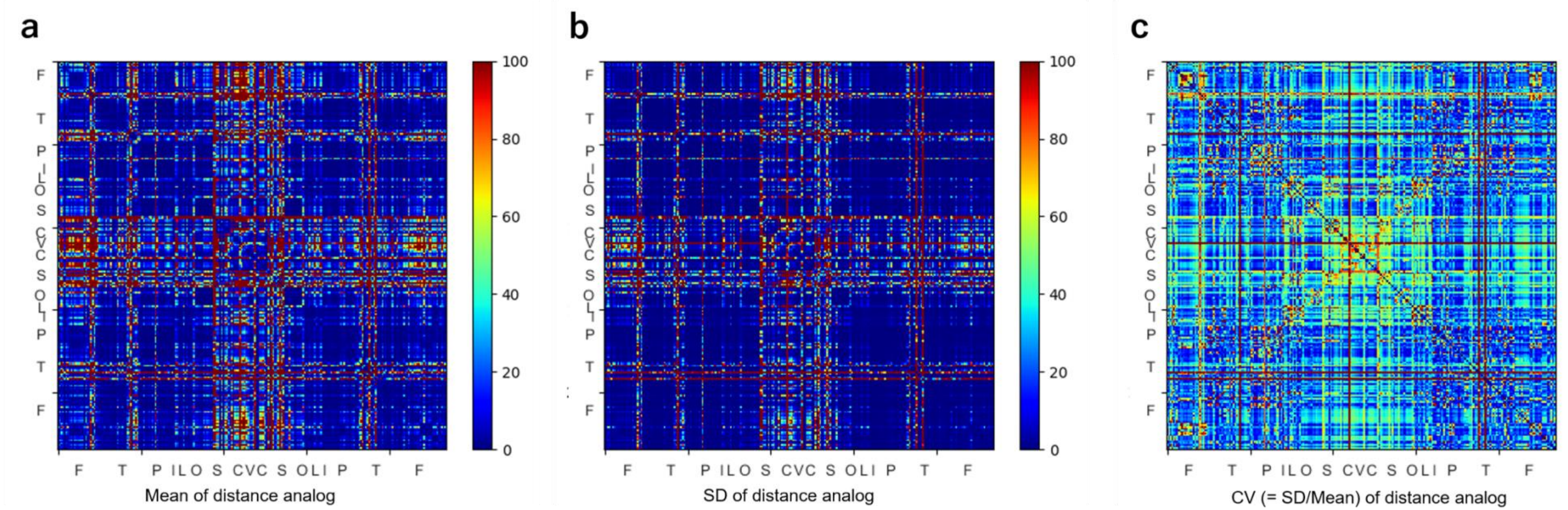

Supplementary Fig. 6 Mean, standard deviation (SD), coefficient of variation (CV) of hyperbolic distance analog of a representative case, which were derived from 100 times-repeated hyperbolic embedding on $\mathbb{S}^1/\mathbb{H}^2$ model. As a descriptive measure of how the hyperbolic length of an edge on embedded hyperbolic disc varies over multiple times of embedding procedure, we used the coefficient of variation (CV) of the hyperbolic distance analog. **a** Mean, **b** standard deviation (SD), **c** coefficient of variation (CV) of distance analog over repetition of embedding is represented in the figure. (Subfigure **c** is same with **Fig. 6b** in the main text but duplicated for the sake of comparability.) Edges with longer distance tended to have bigger variance over repetition of embedding,

and some of them had also larger CV value (**Supplementary Note 2**).

Supplementary Notes

1. Brief description of the geometric $\mathbb{S}^1$/ $\mathbb{H}^2$ model derived from Serrano et al.[1] and Krioukov et al.[2]

A. The similarity space: $\mathbb{S}^1$ model

The similarity space $\mathbb{S}^1$ is a one-dimensional sphere[1], which is equivalent to a circle of radius R. Total number of N nodes are distributed in this circle with density of 1 without loss of generality, so that the radius of circle $\mathrm{R} = \frac{N}{2\pi}$.

N nodes are also assigned a hidden variable, κ, which is proportional to its expected degree. We can assume that the κ and angular position θ are correlated and distributed according to a distribution function of $\rho(\kappa, \theta)$. This kind of model is able to generate community structures and clustering.

If all N nodes are assigned variable $(\kappa_1, \theta_1), (\kappa_2, \theta_2), \cdots (\kappa_N, \theta_N)$, the model propose that each pair of nodes i, j is connected, with probability

$$p_{ij} = \frac{1}{1 + \left(\frac{d_{ij}}{\mu\kappa_i\kappa_j}\right)^{\beta}} \qquad \text{(S2)}$$

where $d_{ij} = \mathrm{R}\Delta\theta_{ij}$ is the arc length between two nodes i and j, with angular distance $\Delta\theta_{ij}$. Two global parameters, μ and β is associated with average degree and global clustering coefficient (or inverse temperature analog), respectively[2,3].

Connection probability can be any integrable function of $\chi = \frac{d}{\mu\kappa_i\kappa_j}$[1], but in this model we implement the Fermi-Dirac distribution model, which defines geometric graphs that are clustered, small-world and with heterogeneous degree distribution.

If we assume that nodes are uniformly distributed along the $\mathbb{S}^1$ space, we obtain the distribution $\rho(\kappa, \theta) = \frac{\rho(\kappa)}{2\pi}$ and if we set $\mu = \frac{\beta}{2\pi\langle k\rangle} sin\frac{\pi}{\beta}$, we obtain expected degree of a node $\bar{k}(\kappa) = \kappa$ and the average degree of network $\langle k\rangle = \langle\kappa\rangle$. This justifies the association of hidden variable κ with the expected degree.

As a result, the process of embedding is equivalent to estimating the $2N + 1$ parameters, $(\kappa_1, \theta_1), (\kappa_2, \theta_2), \cdots (\kappa_N, \theta_N)$ and β.

B. Pure geometric model: $\mathbb{H}^2$ model

The $\mathbb{S}^1$ model can translated into a pure geometric model in the hyperbolic plane, by converting the expected degree κ to the radial coordinate r, by the following:

$$r_i = \hat{R} - 2\ln\frac{\kappa_i}{\kappa_0} \tag{S3}$$

where $\hat{R} \equiv 2\ln\frac{N}{\mu\pi\kappa_0^2}$. Then, the connection probability noted in Eq. (S2) is equivalent to:

$$p_{ij} = \frac{1}{1 + e^{\frac{\beta}{2}(d_{ij}-\hat{R})}} \tag{S4}$$

where

$$d_{ij} \cong r_i + r_j + 2\,ln\frac{\Delta\theta_{ij}}{2} + O\left(\frac{1}{r}\right) \tag{S5}$$

is a good approximation of the distance between two nodes i and j in the hyperbolic space[a]. The hyperbolic law of cosine was used when $\Delta\theta_{ij}$ is very small.

Thus, the connection probability p_{ij} becomes a single-variable function of hyperbolic distance d_{ij}, which makes the model purely geometric. This has important consequences for the global connectivity of network, for instance, the geodesic curves in the hyperbolic plane can be used to efficiently navigate the network[4].

[a] The approximation is accurate for two nodes that are separated by $\Delta\theta_{ij} > \sqrt{e^{-2r_i} + e^{-2r_j}}$

2. Robustness of result, over different choices of threshold

As seen in the (**Supplementary Fig. 1**), we deliberately chose the threshold value in order to maintain scale-freeness while containing most of the nodes in the largest component. Still, as the choice of threshold have small window of arbitrariness caused by subjective assessment by the researcher, it is needed to show the robustness of the result and conclusion of our paper, over slightly different choices of threshold.

A slightly different choice of threshold (namely, around 0.36 for HCP and 0.40 and ABIDE) does show some minor change in the result of embedding (for that the latter includes slightly a smaller number of nodes) but does not change the result in a major way.

We below show results of embedding when threshold value of 0.34/0.36/0.38 on HCP data, and 0.38/0.40/0.42 on ABIDE data is applied for representative cases, each from HCP and ABIDE dataset. Note that the embedding results have arbitrariness of rotation by a constant angle or inversion.

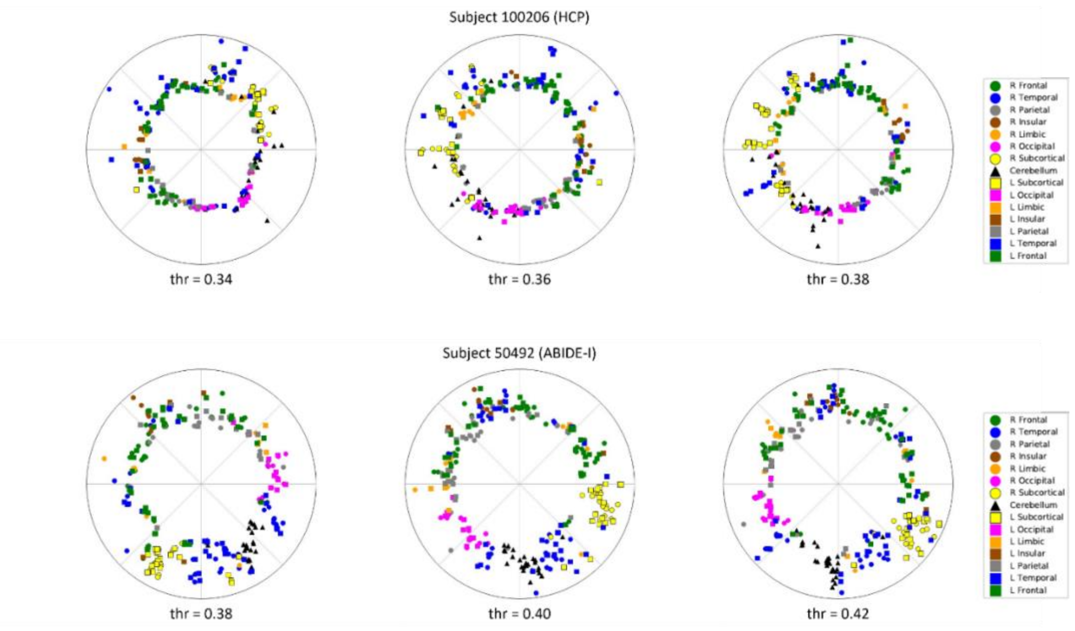

For quantitative assessment of how "angularly similar" the nodes from same anatomic lobes are, we made use of angular coherence ϕ, previously utilized by some researchers[5] in order to describe how certain set of nodes are angularly clustered, defined by the following formula:

$$\xi e^{i\phi} = \frac{1}{N}\sum_{k\in X} e^{i\theta_k} \qquad \text{(S1)}$$

where θ_k is angular coordinate of the point k and N is the number of points in X. As intuitive from Eq. (S1), the higher ξ is, the more points in the set are locally concentrated to form an angularly similar structure.

As we do not have an established statistical criterion to judge how the two embedding results are robust, we descriptively show the angular coherence ϕ of each anatomic lobes among networks from different choices of threshold.

**Subject**	**HCP (100206)**			**ABIDE (50492)**		
**Threshold**	**0.34**	**0.36**	**0.38**	**0.38**	**0.40**	**0.42**
**R Frontal**	0.4690	0.5280	0.4895	0.6109	0.6695	0.7060
**R Temporal**	0.3394	0.3483	0.1318	0.6020	0.3068	0.2850
**R Parietal**	0.3142	0.5215	0.4617	0.6418	0.6382	0.6180
**R Insular**	0.8875	0.8242	0.8505	0.9991	0.9911	0.9960
**R Limbic**	0.4148	0.2956	0.3273	0.2716	0.2034	0.2730
**R Occipital**	0.8520	0.9239	0.8521	0.9793	0.9719	0.9790
**R Subcortical**	0.9327	0.8701	0.8059	0.9881	0.9774	0.9910
**Cerebellum**	0.7669	0.8121	0.8457	0.9780	0.9818	0.9680
**L Subcortical**	0.8273	0.7845	0.8433	0.9356	0.9594	0.9690
**L Occipital**	0.9078	0.9099	0.8801	0.9387	0.9725	0.9830
**L Limbic**	0.4660	0.4751	0.3309	0.3486	0.4041	0.2450
**L Insular**	0.8487	0.8202	0.8253	0.4795	0.7597	0.5920
**L Parietal**	0.3043	0.3948	0.4031	0.7011	0.7092	0.5950
**L Temporal**	0.5021	0.4343	0.3231	0.5342	0.2819	0.3690
**L Frontal**	0.5833	0.5317	0.5694	0.3897	0.5262	0.4550

3. Robustness of result, considering only positively correlated edges

In this work, we have applied the absolute value of Pearson's correlation between brain region, in order to compose the network. This regards the two brain regions with anticorrelated time series as connected, as same as the positive correlation of the same strength. Since the positive and negative correlation between ROIs reflect distinct relationship, we also tried composing the network considering only positively correlated edges.

Please note, the number of negatively correlated edge included in the network after thresholding, is quite small, since the correlation between ROIs are right skewed, as in the figure below, demonstrating distribution of Pearson's correlation.

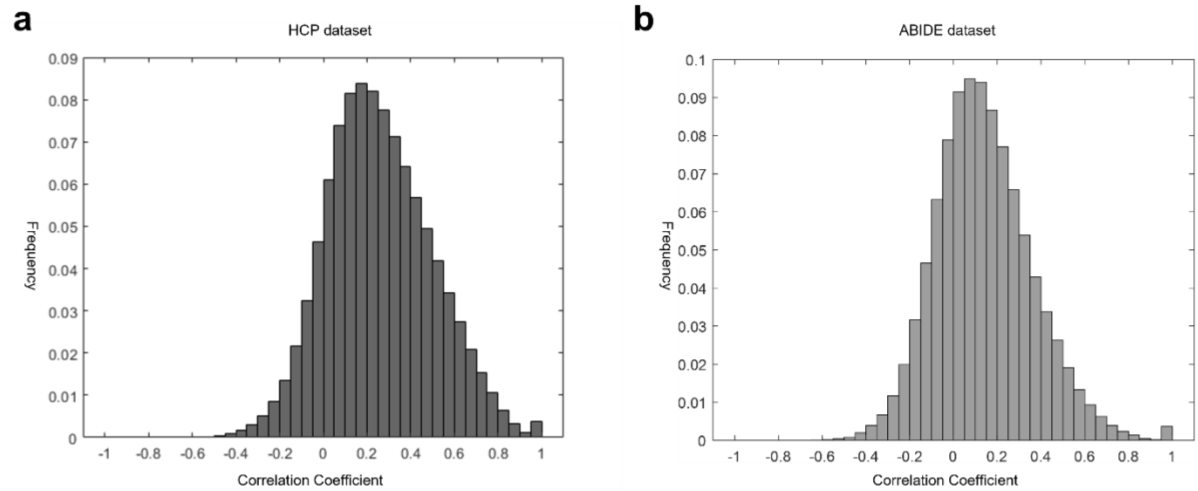

Note that in the work with absolute correlation, we applied threshold value of 0.36/0.40 for HCP and ABIDE, very few negatively correlated edges survive after thresholding.

When we composed the network with only positively correlated edges, the threshold value was determined 0.36/0.40, as same in the case with absolute correlation.

As in the (**Supplementary Note 2**), we have embedded the networks into hyperbolic spaces and compared the networks visually and quantitatively. Note that the embedding results have arbitrariness of rotation by a constant angle or inversion.

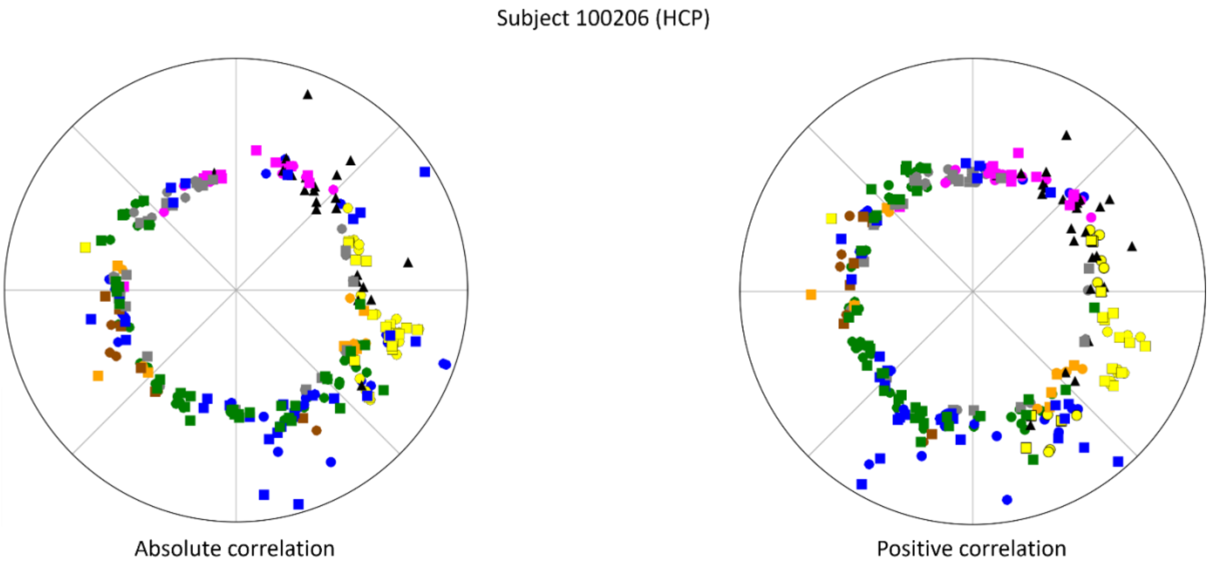

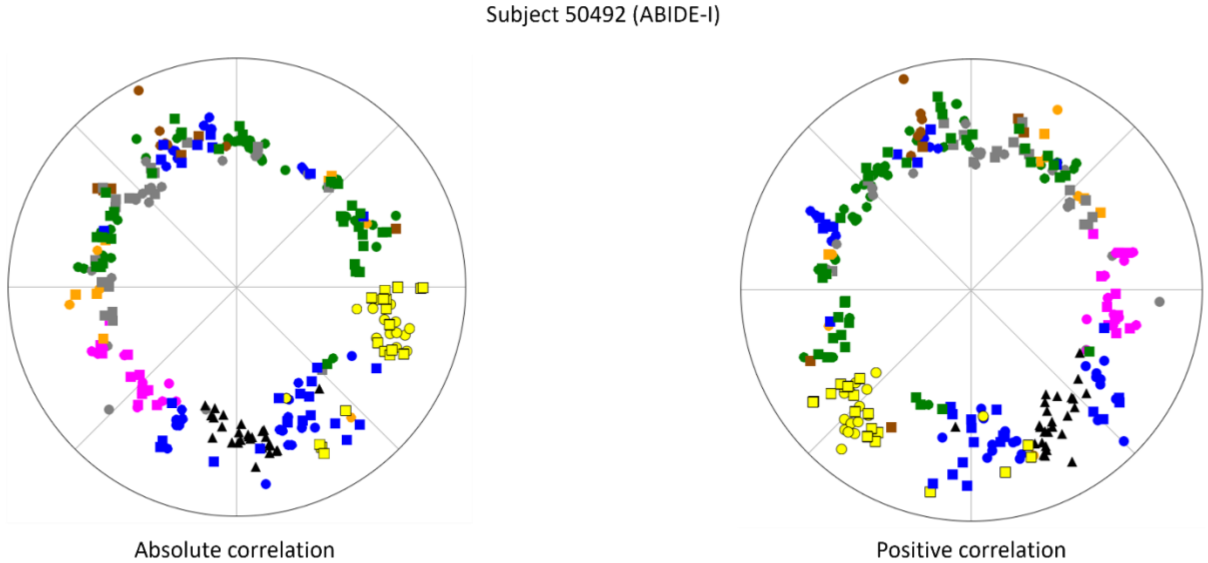

While each pair of embedding results with both positively/negatively correlated edges and only positively correlated edges shows visually similar result, as in the (**Supplementary Note 2**), we computed the angular coherence ϕ defined in Eq. (S1) for each anatomic lobe, in order to quantitatively assess how the nodes from same anatomic lobes are clustered together.

**Subject**	**HCP (100206)**		**ABIDE (50492)**	
**Threshold**	**pos/neg**	**positive**	**pos/neg**	**positive**
**R Frontal**	0.5297	0.4778	0.7268	0.6612
**R Temporal**	0.3123	0.3512	0.4610	0.2958
**R Parietal**	0.4868	0.4426	0.6429	0.6339
**R Insular**	0.8225	0.8377	0.9989	0.9894

**R Limbic**	0.3378	0.3863	0.2644	0.1871
**R Occipital**	0.9262	0.8998	0.9749	0.9733
**R Subcortical**	0.8803	0.9282	0.9751	0.9717
**Cerebellum**	0.8385	0.8584	0.9792	0.9824
**L Subcortical**	0.8020	0.8481	0.9094	0.9298
**L Occipital**	0.9176	0.8581	0.9736	0.9760
**L Limbic**	0.3363	0.3918	0.4916	0.4068
**L Insular**	0.8118	0.7974	0.4483	0.7557
**L Parietal**	0.4125	0.3105	0.8250	0.7121
**L Temporal**	0.4617	0.3831	0.3856	0.2883
**L Frontal**	0.5095	0.5086	0.4693	0.5125

4. Comparison of pattern of distribution between absolute correlation (derived from time-series correlation) and connection probability (after hyperbolic embedding and distance calculation between node pairs)

In this work, we made use of two proximity measures between two nodes. First is the absolute correlation coefficient, which we used to compose the adjacency matrix. Second is the connection probability, which was computed from the hyperbolic distance that resulted from the hyperbolic disc embedding.

Note that the two measures are ranged from 0 to 1, and both get closer to 1 as the two nodes are closely correlated (or functionally similar). However, since the correlation is computed from the quadratic formula of deviation as in Eq. (3) and the connection probability is calculated from the exponential of hyperbolic distance, it is difficult to compare the proximity computed by two measures analytically.

Nevertheless, we can address the similarity of *pattern* of distribution of two proximity measures. To quantitatively assess how similar the two proximity measures are distributed, we computed the root mean square of deviation S, given as the following:

$$\mathrm{S} = \sqrt{\frac{\sum_{i>j}\left|p_{ij} - |\hat{\rho}_{ij}|\right|^2}{N(N-1)/2} - \left|\bar{p} - \overline{|\hat{\rho}|}\right|^2} \tag{S1}$$

where $\bar{p}$ and $\overline{|\hat{\rho}|}$ denotes the mean value of connection probability and absolute correlation over all the edges. N is the number of nodes included in the component, p_{ij} and $\hat{\rho}_{ij}$ denotes connection probability and Pearson's correlation for edges connecting node i and j, respectively. The first term in the square root is the mean square of the edge-wise difference between two measures, while the second term is the difference between mean values of connection probability and absolute correlation value.

Therefore, by Eq. (S1), we can assess the similarity of pattern between two measures, by observing how much larger the mean square of the edge-wise difference between two measures would be than that indicated by the mean difference between two matrices. As S gets smaller, two distributions can be said more similar. Note that S is no less then zero, as easily shown by elementary statistics. The equality of the first and the second term (and thus deviation S = 0) is satisfied when the differences of elements are of constant values over all the edges.

Values of S among 180 subjects from HCP dataset were distributed as Mean = 0.21, SD = 0.062 among subject. For the subjects shown in the (**Fig. 5**) of the main text, the value is 0.18. 0.14, 0.15, and 0.13 for subject 1, 2, 3, and 4, respectively.

When we computed the same measure with the connection probabilities inter-individually among 180 subjects (total of $(180 \times 179) / 2$ pairs), the values of S were distributed as Mean = 0.29, SD = 0.035 among all pairs of subjects. Compared to this inter-individual difference of deviation S values of connection probability between subject pairs, difference of the two proximity measures within individuals had significantly more similar pattern ($P < 0.001$). In this manner, we could address the two proximity measures had the similarity, though not the same, as described in the main text.

5. Coefficient of variation (CV) of repeated hyperbolic embedding and the hyperbolic distance analog between node pairs on this embedded hyperbolic disc

As a descriptive measure of how the hyperbolic length of an edge varies over multiple times of embedding procedure, we used the coefficient of variation (CV), which is a well-known measure of evaluating dispersion, computed by ratio of standard deviation to mean, of the distance analog computed by Eq. (8) in the main text.

$$D_{ij} = e^{\frac{(d_{ij} - \hat{R})}{2}}$$

Though the distance analog is not a linear parameter, the CV measure suffices to compare between edges to evaluate which edge has more aberrant distribution over multiple instances of embedding.

For the representative case same as in (**Fig. 6**) in the main text, we demonstrated the mean, standard deviation (SD) and CV values in (**Supplementary Fig. 4**). Each element of matrix denotes edge. Therefore, the horizontal or vertical line denotes the edges connected to the same node. The red-colored lines shown in the (**Supplementary Fig. 4a**) suggests that all of the edges that are connected to those specific nodes with red elements (edges) have larger hyperbolic distance than others with green/blue/black edges, which means that those red-lined nodes are located farther from most of the other nodes with green/blue/black lines. The edges connected to these nodes also have high standard deviation (**Supplementary Fig. 4b**). And some, but not all, of these lines reappear at the (**Supplementary Fig. 4c**) which suggests higher dispersion meaning non-reproducibility along embedding.

Therefore, we could address, from this case presentation, that some of the nodes that are located on the embedded hyperbolic discs farther from most of the other nodes, which also indicates their non-popularity (very low degree) in functional brain network, have higher arbitrariness over the repetition of embedding.

6. List of abbreviations in Fig. 7

Suffix _R, _L denotes right and left hemisphere.

Frontal lobe

SFG = Superior Frontal Gyrus
MFG = Middle Frontal Gyrus
IFG = Inferior Frontal Gyrus
ORG = Orbital Gyrus
PrG = Precentral Gyrus
PCL = Paracentral Lobule

Temporal Lobe

STG = Superior Temporal Gyrus
MTG = Middle Temporal Gyrus
ITG = Inferior Temporal Gyrus
FuG = Fusiform Gyrus
PhG = Parahippocampal Gyrus
pSTS = Posterior Superior Temporal Sulcus

Parietal Lobe

SPL = Superior Parietal Lobule
IPL = Inferior Parietal Lobule
PCun = Precuneus
PoG = Postcentral Gyrus

Insula Lobe

INS = Insula

Limbic lobe

CG = Cingulate Gyrus

Occipital Lobe

MVOcC = MedioVentral Occipital Cortex
LOcC = Lateral Occipital Cortex

Subcortical Nuclei

Amyg = Amygdala
Hipp = Hippocampus
BG = Basal Ganglia
Tha = Thalamus

Cerebellum and Vermis

CB = Cerebellum
Vermis

References

1 Serrano, M. Á., Krioukov, D. & Boguñá, M. Self-similarity of complex networks and hidden metric spaces. *Physical review letters* **100**, 078701 (2008).

2 Krioukov, D., Papadopoulos, F., Kitsak, M., Vahdat, A. & Boguná, M. Hyperbolic geometry of complex networks. *Physical Review E* **82**, 036106 (2010).

3 García-Pérez, G., Allard, A., Serrano, M. Á. & Boguñá, M. Mercator: uncovering faithful hyperbolic embeddings of complex networks. *New Journal of Physics* **21**, 123033 (2019).

4 Boguna, M., Krioukov, D. & Claffy, K. C. Navigability of complex networks. *Nature Physics* **5**, 74-80 (2009).

5 Osat, S., Radicchi, F. & Papadopoulos, F. k-core structure of real multiplex networks. *Physical Review Research* **2**, 023176 (2020).
